# Nitro-benzylideneoxymorphone, a bifunctional mu and delta opioid receptor ligand with high mu opioid receptor efficacy

**DOI:** 10.3389/fphar.2023.1230053

**Published:** 2023-07-03

**Authors:** Keith M. Olson, Andrea L. Devereaux, Payal Chatterjee, Savanah L. Saldaña-Shumaker, Amanda Shafer, Adam Plotkin, Ram Kandasamy, Alexander D. MacKerell, John R. Traynor, Christopher W. Cunningham

**Affiliations:** ^1^ Department of Pharmacology and Edward F. Domino Research Center, University of Michigan Medical School, Ann Arbor, MI, United States; ^2^ Department of Pharmaceutical Sciences, Concordia University Wisconsin School of Pharmacy, Mequon, WI, United States; ^3^ Department of Pharmaceutical Sciences, University of Maryland School of Pharmacy, Baltimore, MD, United States; ^4^ Department of Psychology, California State University, East Bay, Hayward, CA, United States; ^5^ Department of Medicinal Chemistry, College of Pharmacy, University of Michigan, Ann Arbor, MI, United States

**Keywords:** mu opioid receptor, delta opioid receptor, bifunctional analgesics, tolerance, dependence, SILCS

## Abstract

**Introduction:** There is a major societal need for analgesics with less tolerance, dependence, and abuse liability. Preclinical rodent studies suggest that bifunctional ligands with both mu (MOPr) and delta (DOPr) opioid peptide receptor activity may produce analgesia with reduced tolerance and other side effects. This study explores the structure-activity relationships (SAR) of our previously reported MOPr/DOPr lead, benzylideneoxymorphone (BOM) with C7-methylene-substituted analogs.

**Methods:** Analogs were synthesized and tested *in vitro* for opioid receptor binding and efficacy. One compound, nitro-BOM (NBOM, 12) was evaluated for antinociceptive effects in the warm water tail withdrawal assay in C57BL/6 mice. Acute and chronic antinociception was determined, as was toxicologic effects on chronic administration. Molecular modeling experiments were performed using the Site Identification by Ligand Competitive Saturation (SILCS) method.

**Results:** NBOM was found to be a potent MOPr agonist/DOPr partial agonist that produces high-efficacy antinociception. Antinociceptive tolerance was observed, as was weight loss; this toxicity was only observed with NBOM and not with BOM. Modeling supports the hypothesis that the increased MOPr efficacy of NBOM is due to the substituted benzylidene ring occupying a nonpolar region within the MOPr agonist state.

**Discussion:** Though antinociceptive tolerance and non-specific toxicity was observed on repeated administration, NBOM provides an important new tool for understanding MOPr/DOPr pharmacology.

## 1 Introduction

**FIGURE 1:**
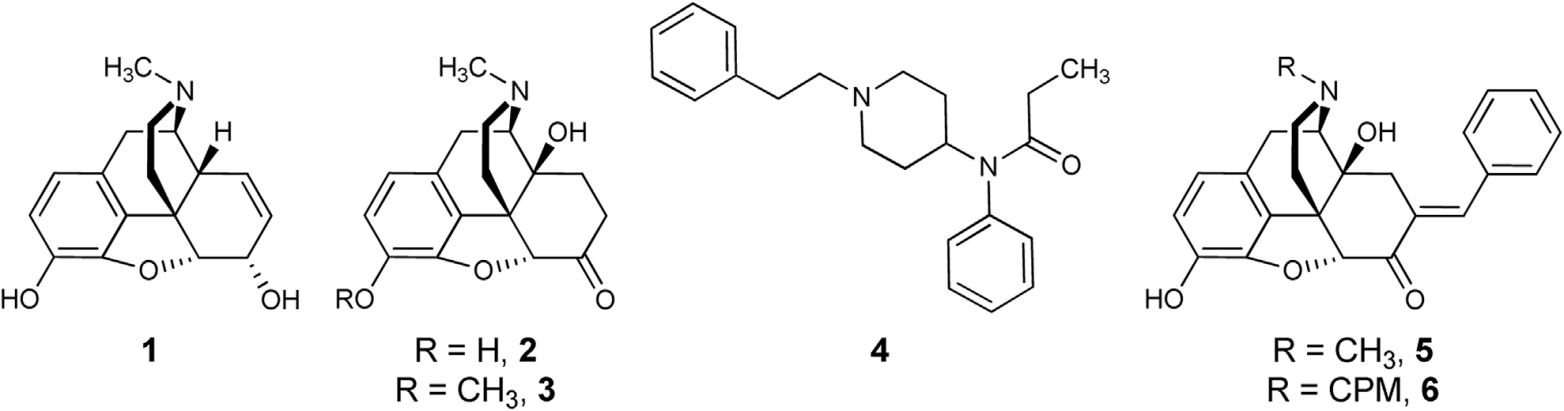
Opioid Analgesics and Bifunctional Lead Scaffolds. Currently available MOPr agonists (morphine, **1**; oxymorphone, **2**; oxycodone, **3**; fentanyl, **4**) and MOPr/DOPr-targeting bifunctional leads (7-*E*-benzylideneoxymorphone, BOM, **5**; 7-*E*-benzylidenenaltrexone, BNTX, **6**.

Chronic pain affected approximately 20% or 50 million adults in the United States in 2016, including 19.6 million who suffered high-impact chronic pain (Dahlhamer et al., 2018). While clinical opioids, including morphine, oxymorphone, oxycodone, and fentanyl (Figure 1), effectively manage moderate-to-severe chronic pain in the clinic (Ballantyne, 2017), serious on-target effects, such as tolerance, dependence, constipation, and life-threatening respiratory depression, are detrimental to their long term use (Holstege and Borek, 2012). For chronic pain patients, continuous opioid use gives rise to analgesic tolerance and the need for escalating opioid doses for the same degree of pain relief. Tolerance to the analgesic actions of opioids develops quicker than tolerance to respiratory depressant effects (Ling et al., 1989), thus increasing the overdose risk and contributing to the ongoing opioid epidemic (Dumas and Pollack, 2008). Therefore, there is a pertinent need to identify analgesics with reduced side effects, such as tolerance, constipation, withdrawal, and dependence.

The on-target analgesic and side effect components of clinical opiates are mediated by activation of the mu-opioid peptide receptor (MOPr) (Burns et al., 2018; Devereaux et al., 2018). MOPrs belong to the opioid peptide receptor (OPr) family of G-protein Coupled Receptors (GPCRs), a family that also consists of the kappa- (KOPr) and delta- (DOPr) opioid receptors. MOPr, DOPr, and KOPr are expressed throughout the brain, spinal cord, and gastrointestinal tract. Critically, the MOPr and DOPr receptors interact with one another and modulate the behavioral effects of one another (Olson et al., 2017).

A plethora of evidence shows that DOPr activity modulates MOPr agonist side effects *in vivo* (Zhu et al., 1999; Ananthan, 2006; Jinsmaa et al., 2008; Olson et al., 2018; Cunningham et al., 2019; Keresztes et al., 2021). Genetic deletion of DOPr prevents the development of tolerance and dependence to MOPr agonists (Zhu et al., 1999), and co-administration of DOPr antagonists attenuates the rewarding effects of MOPr agonists (Abdelhamid et al., 1991). Peptidic and small molecule bifunctional MOPr-agonist/DOPr-antagonists cause less tolerance on repeated administration than selective MOR agonists [e.g. (Schiller et al., 1999; Ananthan, 2006; Healy et al., 2013; Varadi et al., 2016; Olson et al., 2017; Cunningham et al., 2019; Vekariya et al., 2020)]. Key advantages to developing bifunctional MOPr agonist/DOPr antagonists include minimizing the pharmacokinetic and logistical disadvantages of co-administering two drugs (Cunningham et al., 2019).

Early 4,5-epoxymorphinan structure-activity relationships (SAR) studies applied the “message-address concept” to the design of DOPr-selective ligands (Lipkowski et al., 1986; Portoghese et al., 1988). Attaching an aryl function to ring C of oxymorphone mimicked the action of Phe^4^ of leucine-enkephalin and resulted in DOPr-selective antagonists. This led the authors to suggest that this group plays a dual role functioning as part of both the “message” and “address” at DOPr. Aryl rings in this region do not always confer DOPr selectivity, however. For example, adding a 7-position *E*-benzylidene group to the MOPr-preferring agonist **2** results in benzylideneoxymorphone (BOM, **5**), a MOPr partial agonist/DOPr antagonist (Healy et al., 2017; Mada et al., 2020). This is analogous to the work of Portoghese, et al., who first discovered that adding a 7-benzylidene group to the MOPr-preferring antagonist naltrexone results in a MOPr/DOPr antagonist, benzylidenenaltrexone (BNTX, **6**). The fact that the *N*-methyl derivative has higher MOPr efficacy than the equivalent *N*-cyclopropylmethyl (CPM) analog is consistent with the relationship between oxymorphone and naltrexone (Olson et al., 2019). The SARs at DOPr differ from those for MOPr: whereas replacing the *N*-CPM group of **6** with an *N*-methyl resulted in raised MOPr efficacy, the efficacy of **5** at DOPr remained low. This was supported by ligand-based molecular modeling that suggested the basic amine substituent minimally influences the agonist efficacy of 4,5-epoxymorphinans at DOPr (Bernard et al., 2007).

*In vivo* tests confirmed binding and efficacy studies demonstrating that compound **5** is a bifunctional MOPr partial agonist/DOPr antagonist (Healy et al., 2017). In preclinical experiments, compound **5** demonstrated weak, but significant antinociception in mouse hot-plate and tail-flick antinociception tests (Healy et al., 2017), and significant, high-efficacy antinociception in a rat model of inflammatory bladder pain (Terashvili et al., 2021). In rats, **5** showed low abuse liability in self-administration tests (Mada et al., 2020); however, a low level (<20% maximal possible effect or MPE) of antinociception in the warm water tail withdrawal test signifies that the antinociceptive “ceiling effect” of **5** limits its potential effectiveness in treating more severe types of pain.

We conducted a SAR study to determine whether the MOPr efficacy and potency of **5** could be selectively increased while retaining low efficacy at DOPr. Previous SAR studies investigating the benzylidene group of **6** showed that adding substituents to the phenyl ring altered OPr binding affinity and potency but had no impact on efficacy (Bhargava et al., 1997; Palmer et al., 1997). Because the parent compound **6** is a low-efficacy MOPr/DOPr ligand, it is unclear how these or other modifications would influence a higher-efficacy MOPr/DOPr lead. Herein, we report the results of the first SAR study of **5**, preclinical pharmacologic/toxicologic assessment of an improved lead compound, and interpretation of our findings using molecular modeling.

## 2 Results and discussion

### 2.1 Chemistry

**SCHEME 1:**
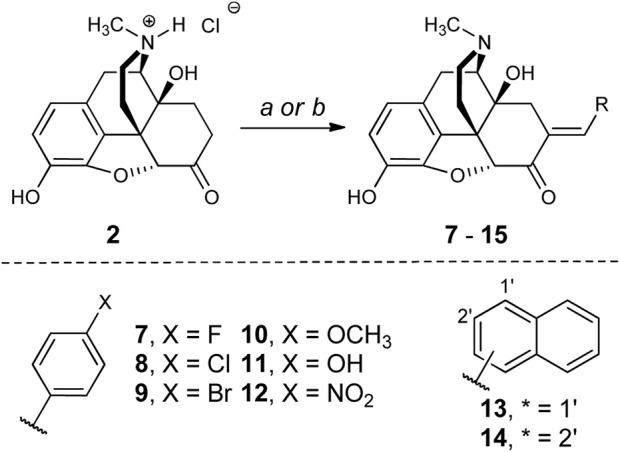
**Reagents and conditions: (a)** RCHO (6 equiv.), piperidine (2 equiv.), MeOH or EtOH, 120°C, 24 h (sealed tube). **(b)** RCHO (6 equiv.), piperidine (2 equiv.), EtOH, 160°C, 1 h (MW).

Microwave-assisted methods were used to generate a series of analogs of 5 (Scheme 1 and Supporting Material). The optimal conditions for the synthesis of **5** called for microwave heating of oxymorphone hydrochloride, benzaldehyde (6.0 equiv.), and triethylamine (2 equiv.) for 1 h in methanol or ethanol at 150°C. Yields were comparable to reflux heating for 24 h (Ohkawa and Portoghese, 1998). These conditions were compatible with electron-rich and electron-poor benzaldehydes. The microwave conditions used to generate mg-scale amounts of analogs were translated toward standard heating methods that could account for higher reaction volumes not possible with the microwave. We noted that microwave-assisted heating caused the internal pressure of the flask to reach 10–12 bar; this pressure was reached uniformly under these conditions and is likely due to the vaporization of the solvent. To replicate these pressures using conventional heating, we conducted the gram-scale synthesis in a sealed tube. After 18 h of heating, both under standard pressure and conditions in a sealed tube, compounds **5** and **12** were generated with similar purities and yields as we observed from microwave methods. The longer reaction times required under these conditions could be due to non-uniform temperature throughout the reaction flask.

The choice of aromatic ring substitutions was deliberate to determine the first SAR of this region of **5**: 1) compounds **7**-**9** test how halogen size and electronegativity contribute to pharmacologic activity; 2) the 4-methoxy (**10**) and -hydroxy (**11**) analogs are electron-donating groups and the 4-nitro analog **12** is electron-withdrawing; 3) the 1- and 2-naphthyl derivatives **13** and **14** are sterically demanding and occupy different chemical space from each other.

### 2.2 *In vitro* pharmacology

The series of R_2_ aryl derivatives of **5** were tested for OPr affinity (K_i_) in competition binding experiments against [^3^H]-diprenorphine using Chinese hamster ovary (CHO) membrane preparations individually expressing MOPr, DOPr, and KOPr (Table 1). Most compounds showed moderate (10–100 nM) to high (<10 nM) affinity at MOPr and DOPr, with preferential binding for MOPr and DOPr over KOPr. Interestingly, **13** (*R*_*2*_ = 1-naphthyl) shows significantly lower MOPr affinity (K_I_ = 75 nM) compared to the regioisomer **14** (*R*_*2*_ = 2-naphthyl: *K*_*I*_ = 1.3 nM) without significantly affecting DOPr affinity (K_I_ = 56 nM and 36 nM, respectively). These results are in contrast to the analogous SAR study with the BNTX **6** scaffold that did not show differences in MOPr affinity between the 1-naphthyl and 2-naphthyl substitutions (Palmer et al., 1997). Compounds **12** and **14** showed the highest affinity at both MOPr and DOPr. All compounds in the series displayed low (Ki > 100 nM) affinity for KOPr.

**TABLE 1:** *In vitro* affinity assessment at MOPr, DOPr, and KOPr. Affinities determined using membrane preparations of CHO cells expressing MOPr, DOPr, or KOPr with [^3^H]-Diprenorphine used as radioligand at MOPr, DOPr, and KOPr. Each *n* run in duplicate, on independent days for *n* = 3. The mean ± SEM of all n’s is reported. ^*a*^Ref(Healy et al., 2017). ^*b*^Ref(Henry et al., 2020). ^*c*^Ref(Olson et al., 2019).

		Ki (±SEM, nM)			Selectivity	
Compound	R	MOPr	DOPr	KOPr	DOPr/MOPr	KOPr/MOPr
**7**	4-F-Ph	19.9 ± 11.7	55 ± 8	>1000	2.8	>50
**8**	4-Cl-Ph	23.8 ± 1.2	47 ± 16	>1000	2.0	>42
**9**	4-Br-Ph	39.9 ± 10.5	121 ± 45	>1000	3.0	>25
**10**	4-OCH_3_-Ph	11.0 ± 2.4	37 ± 9	>1000	3.4	>90
**11**	4-OH-Ph	22.7 ± 6.0	37 ± 7	>1000	1.6	>44
**12**	4-NO_2_-Ph	0.9 ± 0.3	10 ± 3	180 ± 60	11	197
**13**	1-Naphthyl	74.8 ± 24.1	56 ± 20	>1000	0.7	>13
**14**	2-Naphthyl	1.3 ± 0.8	36 ± 4	390 ± 140	28	299
**Naloxone**	N/A	2.8 ± 0.6	49 ± 7	1.7 ± 0.4	17.5	2.4
**5 (BOM**)^***a***^	Ph	17.5 ± 1.1	14.4 ± 0.65	1100 ± 40	0.82	63
**morphine** ^***b***^	N/A	4.2 ± 1.3	104.1 ± 9.1	36.8 ± 9.4	25	8.9
**oxycodone** ^***c***^	H	11 ± 1.8	>2000	>2000	>182	>182

Next, we determined the agonist potency (EC_50_) and efficacy (E_max_) at MOPr, DOPr, and KOPr using [^35^S]GTPγS functional assays in the same membrane preparations as in the binding assays (Table 2). Table 2 shows the range of MOPr potencies between 2.2 ± 0.5 nM (**12**) and >1,000 nM (**8**, **9**, and **13**). Again, compounds **12** and **14** showed the highest MOPr potency. The MOPr potency of **12** was approximately 10-fold higher than that reported for oxymorphone (Olson et al., 2019). Compared to **5**, all compounds showed higher MOPr efficacy, ranging from 69% to 87% E_max_, with comparable E_max_ to oxymorphone. **12** and **14** showed higher affinity, potency, and efficacy than **5** at MOPr, despite their R_2_ substituents being dissimilar: electron-withdrawing NO_2_ group for **12** and sterically demanding 2-naphthoyl group for **14**. The increased MOPr efficacy observed in the present *N*-methyl series is unique compared to prior SAR studies, where arylidene substitutions in the *N*-17-CPM and -allyl analogs resulted exclusively in MOPr antagonists.

**TABLE 2:** *In Vitro* functional activity assessment at DOPr, KOPr, and MOPr. [^35^S]GTPγS binding was performed in the same membrane preparations as the binding assays. E_max_% calculated using DAMGO, U69,593, and DPDPE as standard agonist ligands, at MOPr, KOPr, DOPr, respectively. Each n run in duplicate, on independent days for *n* = 3. The mean ± SEM of all *n*’s is reported. ND = Not Determined. DNS = Did Not Stimulate. ^*a*^Ref (Healy et al., 2017). ^*b*^Ref(Henry et al., 2020). ^*c*^Ref (Olson et al., 2019).

	MOPr		DOPr		KOPr		Selectivity
Compound	EC50 ± SEM (nM)	Emax ±SEM (nM)	EC50 ± SEM (nM)	Emax ±SEM (nM)	EC50 ± SEM (nM)	Emax ±SEM (nM)	DOPr/MOPr
**7**	95 ± 25	69 ± 16	ND	<15%	ND	ND	ND
**8**	>1000	ND	145 ± 14	9 ± 3	ND	ND	<0.15
**9**	>1000	ND	ND	<15%	ND	ND	ND
**10**	172 ± 55	80 ± 5	102 ± 8	30 ± 9	ND	ND	0.59
**11**	275 ± 100	85 ± 6	97 ± 40	21 ± 6	ND	ND	0.35
**12**	2.2 ± 0.5	87 ± 7	20 ± 5	28 ± 4	ND	<15%	9.1
**13**	>1000	ND	102 ± 37	36 ± 10	ND	ND	17
**14**	12 ± 5	78 ± 11	ND	<15%	ND	<15%	ND
**DAMGO**	36 ± 5	100	ND	ND	ND	ND	ND
**Deltorphin-II**	ND	ND	77 ± 15	100	ND	ND	ND
**U69,593**	ND	ND	ND	ND	4.3 ± 0.4	100	ND
**morphine** ^***b***^	147 ± 48	98.5 ± 3.8	>593	>30	DNS	DNS	>4
**5 (BOM)**	63 ± 25	53 ± 9	26 ± 4	22 ± 4	ND	ND	0.41
**oxycodone** ^***c***^	23 ± 5	98 ± 6	>2,000	ND	>2,000	ND	>87

Measurable DOPr potencies ranged from 20 nM (**12**) to 145 nM (**8**) and consistently showed weak partial agonist efficacy. Compounds **12** and **14** emerged as candidates with high MOPr agonist potency and potential DOPr antagonist activity*.* In each case, the DOPr/MOPr selectivity ratio was approximately 1:10, with relative potency rank order of **12** > **14**. We further evaluated **12** in βarrestin2 recruitment assays (DiscoveRx) to evaluate DOPr agonist vs. antagonist efficacy in a higher efficacy requiring assay (Supplementary Figure S1). Compound **12** alone did not stimulate DOPr-mediated βarrestin2 recruitment at any concentration but produced a rightward shift of the SNC80 dose-response curve at 10, 100, and 1,000 nM (K_B_ = 32 nM ± 11). These results are consistent with observations that G-protein assays are often more sensitive to partial agonists than arrestin recruitment. None of the compounds in this series stimulated KOPr-mediated G-protein recruitment. Retaining selectivity over KOPr is vital because KOPr agonists cause dysphoria and hallucinations (Bruchas et al., 2007; Cunningham et al., 2011).

Some general SAR trends were observed. In the halogen series (**7**-**9**), we noted that MOPr and DOPr affinity generally decreased as a function of increasing halogen size and decreasing electronegativity. In the functional assays, only the *para*-fluoro derivative **7** displayed weak MOPr partial agonism. We also noted only minor pharmacodynamic differences between the 4-methoxy and hydroxy analogs (**10** and **11**). When compared to the parent compound **5**, these electron-rich derivatives were lower-potency, higher-efficacy MOPr agonists that maintained a similar DOPr/MOPr selectivity profile. The strong electron-withdrawing derivative **12** showed the highest affinity of MOPr and DOPr in the series. When comparing to the halogen series, the fact that the strongest OPr activity was seen with the most electronegative and strongest electron-withdrawing groups suggests that electronegative functional groups are preferred in this space. The striking difference between MOPr activity for **13** (1-naphthyl) and **14** (2-naphthyl) suggests that the aromatic binding site has low tolerance for sterically demanding groups fused to the *ortho*- and *meta*-positions, but higher tolerance for *meta*/*para*-substitution.

Based on these initial results, we opted to advance compound **12** (termed nitro-BOM, or NBOM) toward *in vivo* behavioral tests. Although 12 did show some degree of DOPr stimulation in the [35S]GTPγS assay, low-efficacy DOPr partial agonists frequently act as antagonists *in vivo* when the endogenous DOPr system is active, and/or do not possess sufficient agonist efficacy to produce agonist behaviors *in vivo* at DOPr.[e.g., (Dripps et al., 2020)^,^ (Broom et al., 2002)] Furthermore, the low partial agonist activity of compound **12** is not expected to alter the desired therapeutic profile–analgesia with reduced tolerance–since MOPr agonists with DOPr agonist or antagonist activity can produce similar (though not identical) profiles (Olson et al., 2017).

### 2.3 *In vivo* pharmacology

#### 2.3.1. Acute antinociception

**FIGURE 2:**
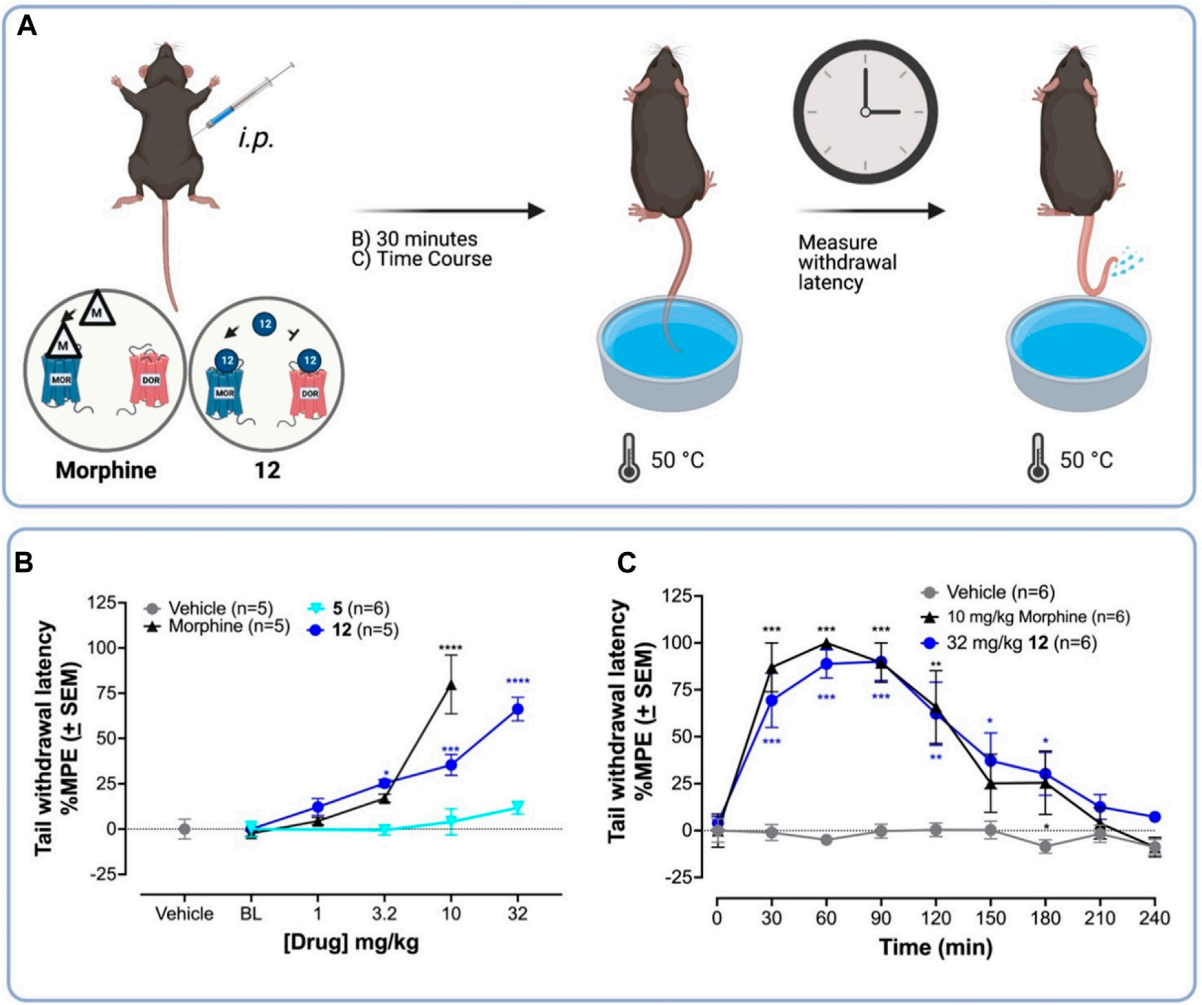
**12** produces higher antinociceptive activity than the parent **5** in the WWTW assay in C57BL/6 mice. **(A)** Procedure for warm water tail withdrawal mouse tail-flick antinociceptive assay. Vehicle or drug was injected *i. p.* into naïve C57BL/6 mice followed by testing for antinociception—via measuring the tail withdrawal latency time—using 50°C warm water. **(B)** Dose-response curves for antinociception of **12**, the parent compound **5**, the standard MOPr agonist morphine, and vehicle. **12** is approximately 3-fold less potent than morphine in the WWTW antinociception assay. **(C)** Time course of antinociception following i. p*.* Administration of 10 mg/kg morphine—a standard MOPr agonist—and 32 mg/kg **12** both producing near-maximal latency. A one-way ANOVA to compare each dose of **5**, **12,** or morphine treatment to vehicle showed a significant effect of the drug dose on tail withdrawal latency [F (13,60) = 18.9; *p* < 0.001]. Post hoc comparisons using the Dunnett’s multiple comparisons test indicated that 10 mg/kg morphine, and 3.2 mg/kg, 10 mg/kg, and 32 mg/kg **12** significantly increased %MPE tail withdrawal latency. ****p < 0.0001; ***p < 0.001; **p < 0.01; *p < 0.05.

**FIGURE 3:**
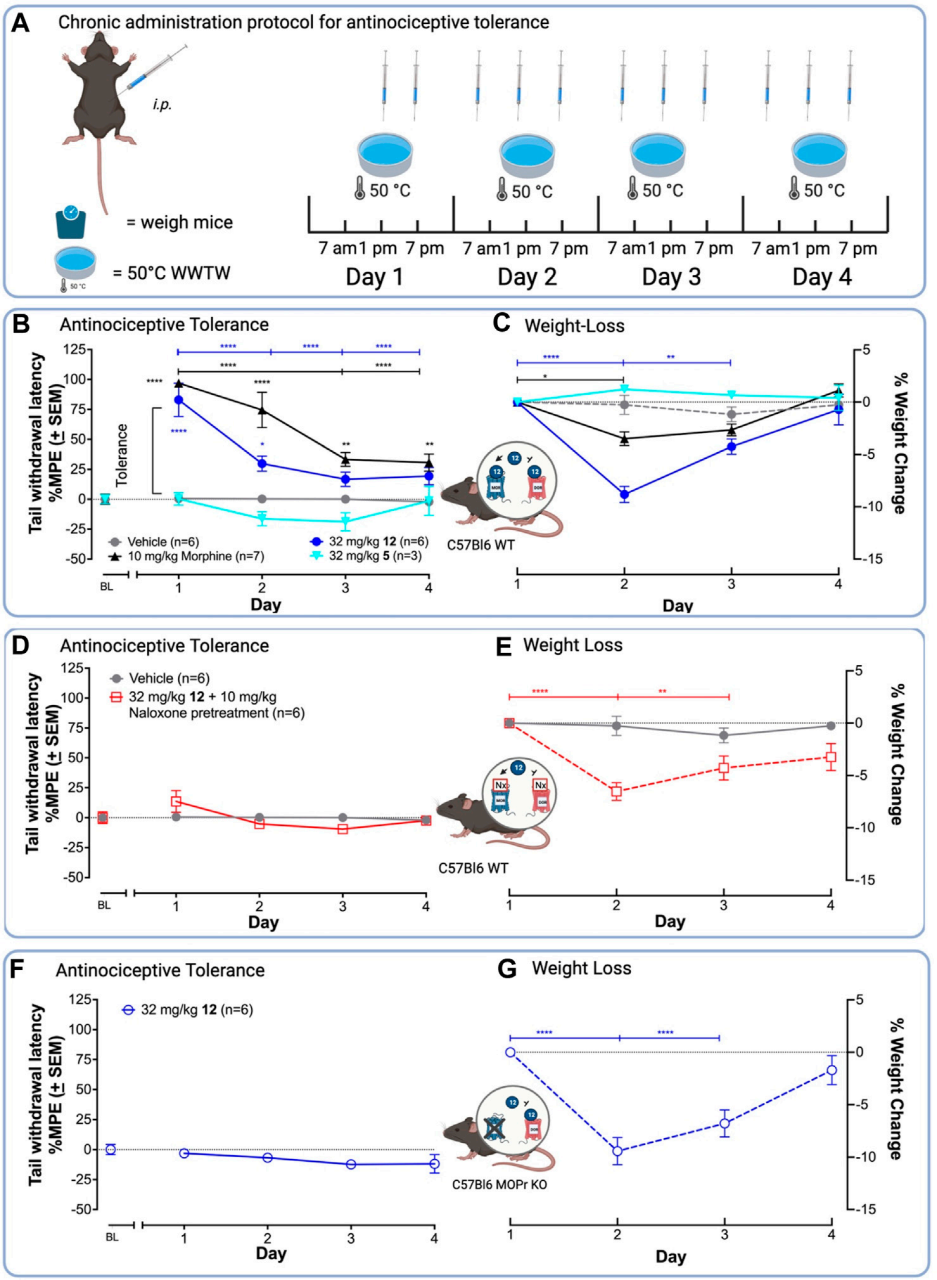
**12** produces antinociception and antinociceptive tolerance mediated by MOPr in the WWTW assay. **(A)** Chronic administration paradigm for the antinociceptive tolerance experiment using the 50°C WWTW assay. Vehicle or drug was injected i. p. into naïve C57BL/L6 mice 3 times daily for up to 5 days followed by once-daily antinociception testing**. (B) 12** in the presence of saline produces similar antinociceptive tolerance to morphine after chronic administration with both producing minimal antinociception by Day 3. **(C)** Mice treated with **12** lost significantly more weight than mice treated with morphine. **(D and E)** Pretreatment of C57BL/6 WT mice with 10 mg/kg naloxone before administration with 32 mg/kg **12** blocked antinociception **(D)** but not weight loss **(E)**. **(F and G)** Treatment of C57BL/6 MOPr KO mice with 32 mg/kg **12** blocked antinociception **(F)**, but did not alter weight loss induced by **12 (G)**. Insets: Arrow indicates activation; the bar indicates inhibition. A two-way ANOVA showed significant differences between days [F (5, 115) = 21.05, *p* < 0.0001] and drug treatment [F (4, 23) = 98.94, *p* < 0.0001]. Statistically significant differences in the follow-up *post hoc* Tukey analysis are indicated between days with *s and brackets in the drug color. Antinociception within drug groups is compared to baseline (BL) for drugs with * in the drug color. ****p < 0.0001, ***p < 0.001, **p < 0.01, and *p < 0.05.

The 50°C warm water tail withdrawal (WWTW) antinociception assay is a standard preclinical pain assay, in which clinical analgesics increase the latency of tail removal from the warm water (Figure 2A). (Negus, 2019) C57BL/6 wild-type (WT) mice were treated with the vehicle, or increasing concentrations of morphine, **5** (the parent compound), or **12** (the new lead) *i. p.* 30-min prior to WWTW testing. Figure 3B shows that compound **12** produces a dose-dependent increase in tail withdrawal latency up to 34 mg/kg. The parent compound **5** produces negligible antinociception over this range, which is consistent with its low MOPr efficacy.

Next, we compared the antinociceptive time-course of **12** and morphine in the 50° WWTW assay. C57BL/6 mice were administered (*i.p.*) 32 mg/kg **12** or 10 mg/kg morphine followed by antinociception testing every 30 min until responses returned to baseline (Figure 2C). A two-way ANOVA showed 10 mg/kg morphine and 32 mg/kg **12** significantly increased tail withdrawal latency compared to vehicle [F (2,12) = 10.7; *p* = 0.0001]. Post hoc comparisons indicated that 32 mg/kg **12** and 10 mg/kg morphine significantly increased tail withdrawal latency at 30 and 60 min, compared to vehicle (*p* < 0.05). To compare the total antinociceptive activity over time, we determined the area under the curve (AUC) of each ligand’s time-course. An unpaired *t*-test to compare the AUC of morphine (M_AUC_ = 11,934 s*min ±1817) and **12** (M_AUC_ = 9247 s*min ±1765) revealed no significant difference between the two treatments [*t* (10) = 1.06, *p* = 0.32]. These doses were used to compare the development of tolerance in subsequent experiments.

#### 2.3.2 Chronic tolerance

To test the hypothesis that MOPr/DOPr ligands produce less tolerance than selective MOPr agonists (Shen and Crain, 1995; Yamazaki et al., 2001; Lowery et al., 2011; Mabrouk et al., 2012; Stevenson et al., 2015; Lei et al., 2019), we compared the ability of a standard clinical MOPr agonist, morphine, and the lead **12** to produce antinociceptive tolerance upon chronic administration (Figure 3A). Mice were injected with drug 3x daily, for 4 days and tested for antinociception once daily. In this assay, tolerance corresponds to a decrease in tail withdrawal latency after multiple drug exposures. In C57BL/6 WT mice, 10 mg/kg morphine and 32 mg/kg **12** showed a significant decrease in antinociception on days 3 and 4 (Figure 3B), with the %MPE tail-withdrawal latency falling from –80 to 90%–25% in both cases. Unexpectedly, 32 mg/kg **12** produced tolerance by day 2, as indicated by a significant difference in tail-withdrawal latency between day 1 and day 2, whereas morphine did not show statistically significant tolerance until day 3.

To demonstrate antinociception was mediated by MOPr, we repeated the chronic administration experiments in WT C57BL/6 mice pretreated with the MOPr-preferring antagonist, naloxone, or in C57BL/6 mice lacking the MOPr (MOPr KO). Pre-administration with naloxone blocked antinociception (Figure 3D). Similarly, MOPr KO mice treated with 32 mg/kg **12** did not show antinociception, indicating that MOPr agonist activity is required for antinociception (Figure 3F). As expected, the OPr antagonist and MOPr KO experiments indicate that antinociception and likely tolerance are MOPr-mediated effects.

The current work suggests that a bifunctional MOPr/DOPr pharmacodynamic profile alone is insufficient to produce antinociception with reduced tolerance. Other studies support this. For example, SoRI 9409 showed antinociception and minimal tolerance in the low efficacy-requiring acetic acid writhing test (Wells et al., 2001), but was inactive in the WWTW test. In another set of experiments evaluating a series of MOPr agonist/DOPr antagonists (AAH8, AMB46, and AMB47), morphine and AMB46, but not AAH8 or AMB47, produced tolerance using a twice-daily escalating dose regime (Anand et al., 2018). This demonstrates that diminished antinociceptive tolerance is not a characteristic of all bifunctional MOPr agonist/DOPr antagonist ligands.

A possible explanation of findings reported here is that the MOPr/DOPr binding profile of **12** (DOPr/MOPr selectivity = 11, Table 1) is not “balanced” enough for DOPr antagonism to counteract MOPr agonism; however, other bifunctional MOPr/DOPr ligands, such as UMB 425 (DOPr/MOPr affinity ratio >60), are similarly “unbalanced” and yet still show attenuated development of tolerance (Healy et al., 2013). Future studies to determine the mechanistic reasons for these differences are important to consider in order to retain the desired therapeutic profile during lead optimization of MOPr agonist/DOPr antagonist ligands. Potential mechanistic inquiries include evaluating the possibility of MOPr/DOPr heterodimerization (Olson et al., 2021), downstream cross-talk in the same neuron, on neural circuits, and pharmacokinetic (PK) differences (e.g., protein binding, blood-brain barrier permeability, and plasma exposure).

#### 2.3.3 Toxicology

Mice treated with 32 mg/kg **12** during the tolerance experiments showed equal or greater weight-loss than those treated with morphine, a common adverse effect of MOPr agonists in mice associated with constipation and nausea (Figure 3C). (Bomzon, 2006; Jirkof et al., 2015; Jirkof, 2017) Preliminary experiments showed mice treated with 32 mg/kg **12** became sick and had to be euthanized; mice administered morphine did not require euthanasia, indicating MOPr agonism alone cannot explain this observation. Subsequent dissection revealed severe GI blockade indicating constipation. Consequently, for the chronic treatment experiments with **12**, mice were supplemented with 0.5 mL saline (*s.c.*) twice daily. This improved health and no animals had to be euthanized during the experiment. Due to these observations and that both MOPr and DOPr agonists are known to induce constipation (Galligan and Sternini, 2017), we aimed to examine if the weight-loss was an off-target or on-target action of **12**. Chronic administration of 32 mg/kg **12** *i. p.* in C57BL/6 MOPr WT pretreated with naloxone (10 mg/kg) (Figure 3E) and C57BL/6 MOPr KO mice (Figure 3G) led to a 5%–10% loss of body weight by day 2, comparable to C57BL/6 WT animals (Figure 3C), indicating weight-loss does not require MOPr.

Finally, to determine if the weight loss observed for **12** was common among the class of 7-benzylidene-substituted oxymorphones, we repeated the tolerance experiments with the parent compound **5** (32 mg/kg). Compound **5** produced neither weight-loss nor antinociception in C56BL/6 WT mice (Figures 3B,C), suggesting the weight-loss induced by **12** is due to the presence of the *para*-NO_2_ group. This group of **12**, which is absent in **5**, is a common toxicophore that may be reduced *in vivo* into reactive metabolites (Nepali et al., 2019), or could activate the olefin to make it a stronger electrophile. On the other hand, other MOPr/DOPr ligands did show weight loss in preclinical rodent models (Anand et al., 2018), suggesting that perhaps a common mechanism underlies these toxicities. Future studies are needed to delineate the source of this toxicity before analgesics of this class can progress toward clinical development.

### 2.4 Computational modeling

To further understand the *in vitro* SAR of analogs of **5** at MOPr and DOPr, we used the computational modeling technique, Site Identification by Ligand Competitive Saturation (SILCS) (Raman et al., 2011; Raman et al., 2013; Yu et al., 2014; Faller et al., 2015; Yu et al., 2015; Raman et al., 2017; Ustach et al., 2019), to elucidate predicted atomic-level SAR detail between the receptor and ligands. SILCS is a functional-group mapping approach that uses Grand Canonical Monte Carlo/Molecular Dynamics (GCMC/MD) simulations to sample the distribution of water and small solutes around a protein as well as explicit treatment of protein flexibility. The approach utilizes multiple small solutes representing different functional groups to generate functional group affinity maps, called FragMaps, encompassing MOPr and DOPr. The SILCS FragMaps may be used to calculate approximate binding affinities of the ligands, called ligand grid free energies (LGFE), as well as determine the free energy contribution of each classified atom to the overall binding affinity. Using the SILCS FragMaps, we docked the analogs of **5** to estimate their relative binding free energies to both MOPr and DOPr and to understand how ligand functional groups contribute to the relative affinities.

Using SILCS-Membrane simulations, we generated FragMaps for the active (PDB ID: 5C1M) (Huang et al., 2015) and inactive MOPr (PDB ID: 4DKL) (Manglik et al., 2012) crystal structures, and active [modelled from PDB ID 6PT3 (Claff et al., 2019)] and inactive DOPr [PDB ID: 4EJ4 (Granier et al., 2012)] with eight solutes representing different chemical functionalities. The resulting FragMaps for ligand docking and LGFE scoring are comprised of 1) apolar maps based on benzene and propane; 2) generic hydrogen bond donor (GEND) maps using the N of formamide and N(H) of imidazole; 3) generic heterocycle carbon (GEHC) maps using the carbon atoms of imidazole; 4) generic hydrogen bond acceptor (GENA) maps using the O of formamide and acetaldehyde, and N imidazole; and individual, specific maps based on the 5) methanol O (MEOO), 6) methylammonium N (MAMN, positive), and 7) acetate carbonyl C (ACEC, negative) atoms.

**FIGURE 4:**
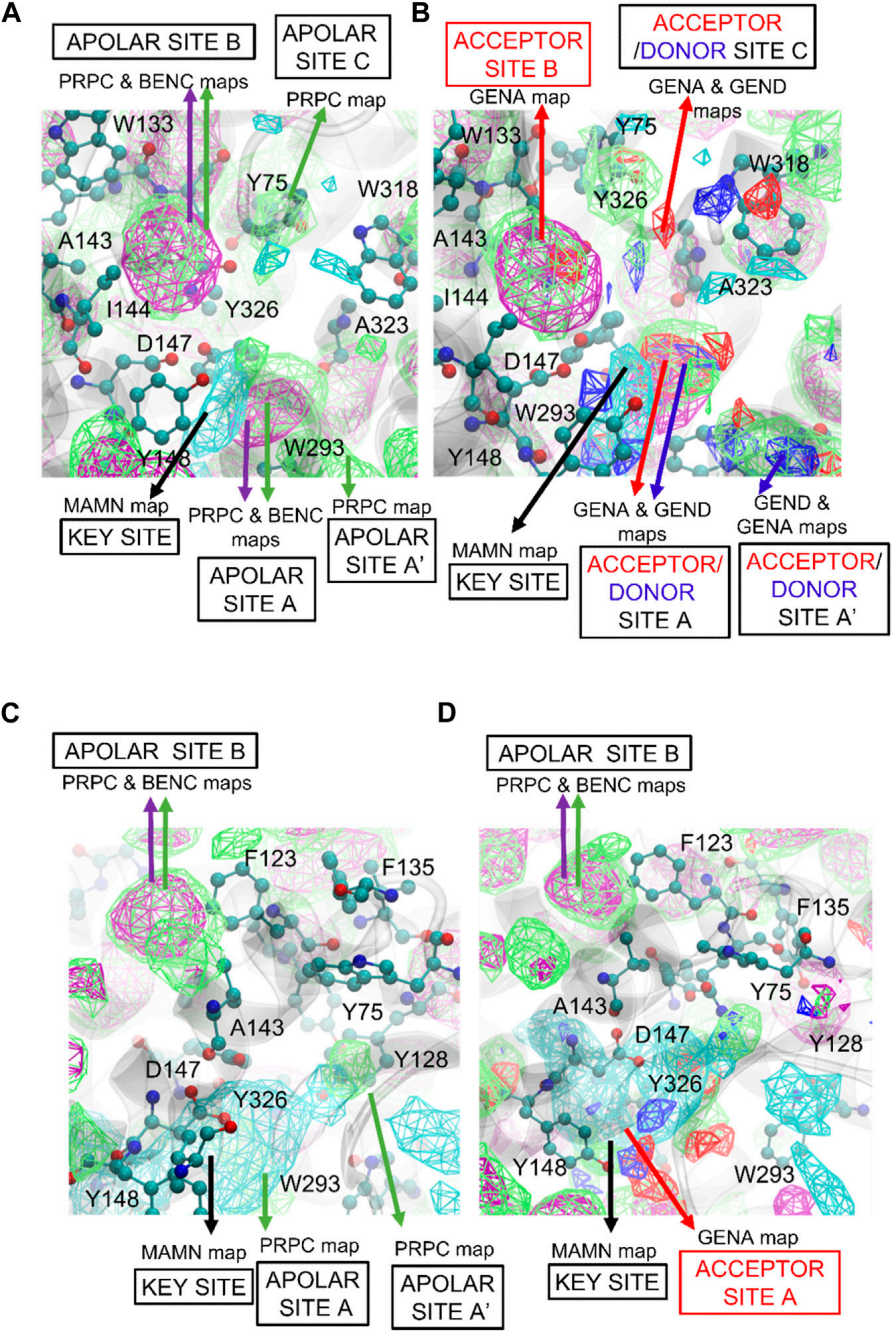
SILCS FragMaps overlaid on the **(A and B)** active and **(C and D)** inactive crystal structures of MOPr. The protein backbone is shown in a transparent gray cartoon with selected sidechains shown in atom colored CPK format. FragMap Color Code: Benzene (purple), Apolar (green), positive (cyan) at GFE energy contours of −1.2 kcal/mol.

FragMaps were generated for both the active and inactive forms of the MOPr and DOPr receptors to differentiate energetic contributions to the binding of agonists and antagonists, respectively (Figure 4 and Supplementary Figure S2). Figure 4 represents both active (Figures 4A,B) and inactive (Figures 4C,D) pockets of MOPr, where apolar and positive FragMaps are shown in Figure 4A for active and Figure 4C for inactive forms. As expected, positively charged maps (MAMN, cyan) are near D147 in both active and inactive conformations. The positive MAMN maps formed near the key residue D147 of TM3 is labeled as the “key site”. However, the apolar aromatic (BENC) and aliphatic (PRPC) maps (green and purple meshes, respectively) are different between the active and inactive conformations. The active pocket has three distinct apolar sites: A, B, and C (Figure 4A,B), labelled according to their proximity to the key site near D147. The respective extensions to these sites further from the key site are labelled as sites: A′ and B’. Apolar site A is located by residues W293 of TM6 and Y326 of TM7 closer to the key site, apolar region B is located near amino acids F123, Q124, W133, V143, and I144, and apolar region C is near amino acids Y75 and Q124. The side chain of Q124 is positioned between apolar site B and C. While apolar site B contains both aromatic and aliphatic apolar maps, site C is primarily composed of small aliphatic apolar maps in both active and inactive pockets (Figures 4A–D). As depicted in Figures 4B,D, both active and inactive pockets also contain hydrogen-bond acceptor and donor sites that overlap with the apolar sites. While the active MOPr pocket is composed of large favorable acceptor and donor regions, the regions are much smaller in the inactive pocket with site B no longer present in the inactive MOPr. Notably, the positive (MAMN) maps formed closer to the key residue D147 in the active versus the inactive MOPr structure. Additionally, the positive key site in the inactive pocket was larger than the active pocket, in agreement with the well-known expanded form of the pocket in inactive MOPr structures (Dripps et al., 2020) indicating that qualitative differences in the functional groups distributions as seen in the FragMaps may drive ligand binding to the active vs inactive forms of the receptor. Similar differences in active and inactive DOPr pockets are present as detailed in the (Supplementary Figure S2 and associated text).

**FIGURE 5:**
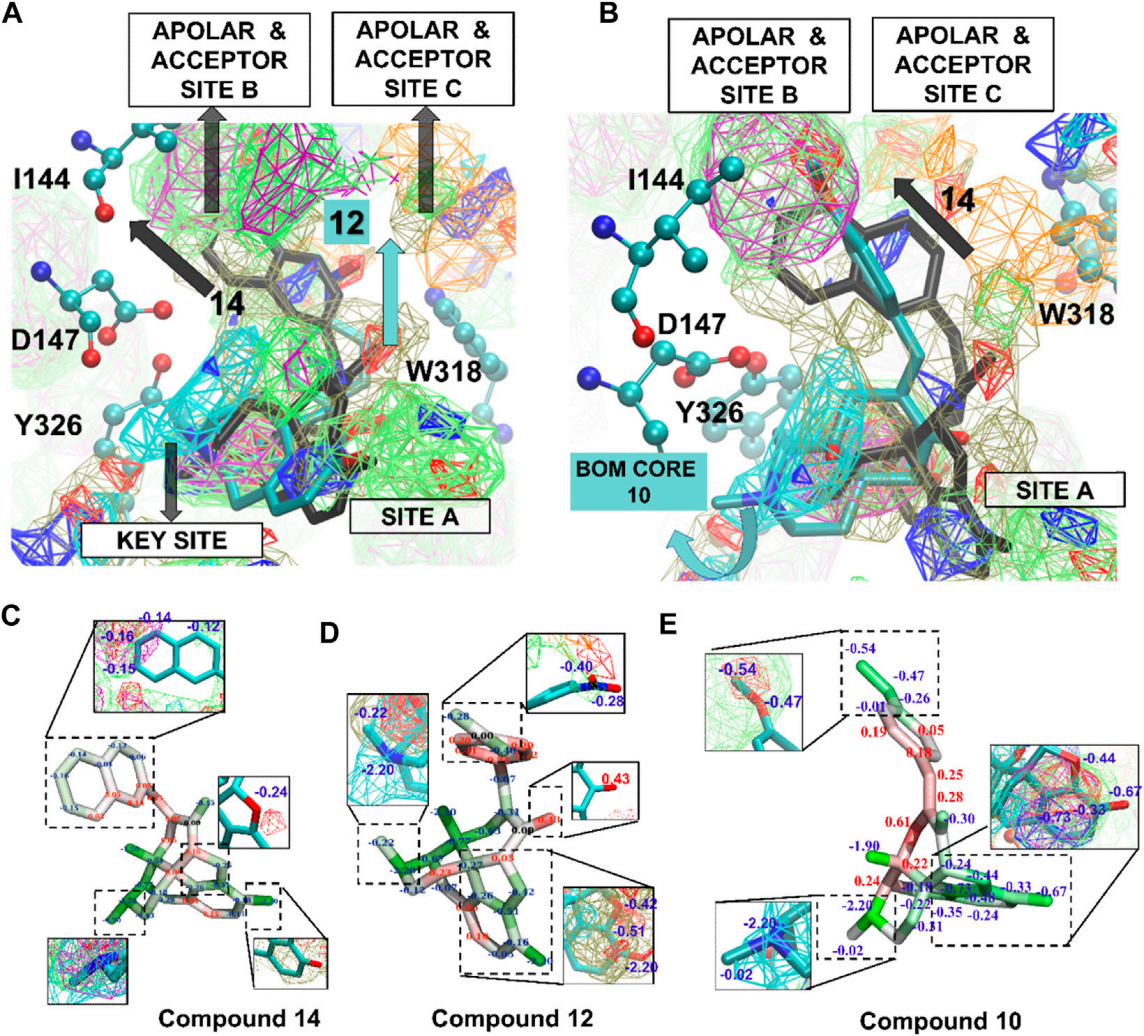
**(A)** SILCS-MC poses of compounds **12** and **14** in the active receptor pocket of MOPr. Amino acid side chains are represented in atom colored CPK and ligands in licorice representation. **(B)** SILCS-MC poses of **14** and **10** in the active receptor pocket. Curved blue arrow represents a displaced orientation of the 4,5-epoxymorphinan core (BOM core) of **10** from the apolar site **(A,C,D and E)** Compounds **14**, **12** and **10** represented in licorice representation with atom based GFE (blue letters—favorable GFE, red letters—GFE based penalties). FragMap Color Code: Benzene (purple), propane (green), methylammonium N positive (cyan), generic hydrogen-bond acceptor (red), generic hydrogen-bond donor (blue), methanol oxygen (olive green), and acetate O negative (orange) at GFE energy contours of −1.2 kcal/mol.

Figure 5 shows the SILCS-MC docked poses for **12**, **14**, and **10** in the active MOPr pocket, with the N17 basic amine overlaid on the MAMN map key sites. These three compounds were the top compounds as per the *in vitro* experiments with their experimental binding affinities as **12** > **14** > **10**. On the other hand, the top three compounds as per their LGFE scores were **14** > **9** = **7** > **12** (Table 3), thus aligning the two of the three top scoring ligands with their *in vitro* ranks. Figure 5A shows the SILCS-MC poses of **12** and **14**, where the nitro group of nitrophenyl of **12** orients itself close to the acceptor site C, which displaces its core from apolar site A and nitrophenyl from apolar site B. Such a displacement thus incurred penalties on the molecule due to a lack of overlap with apolar FragMaps (BENC or PRPC), thus altering its *in silico* ranking (Figure 5C,D). Figure 5B compares the SILCS-MC poses of **14** and **10**, showing that the 4,5-epoxymorphinan core of **10** rotates to displace the compound from apolar site B, thus also incurring penalties on the phenyl of the methoxyphenyl ring. Figures 5C–E shows **14**, **12** and **10** with their GFE scores, where the important parts of each ligand have been magnified to show the overlap with the respective FragMaps. As depicted in Figure 5C, the 2-napthyl head group of **14** partially occupied apolar site C, incurring penalties on multiple carbon atoms in the napthyl group, while the 4,5-epoxymorphinan core remained seated in apolar site A, with the basic amine most ideally located in the key site. Such a location of **14** proved rewarding in terms of the LGFE scores, where compound **14** was the most favorable (−10.12 kcal/mol), while the nitro-phenyl group of **12** incurred penalties at the phenyl ring due to a lack of overlap with apolar FragMaps at site C (Figure 5D). Similarly, **10** incurred a less favorable score due to a lack of overlap of some atoms of the methoxyphenyl group, with apolar site C (Figure 5E). The LGFE scores of **9** and **7** were almost equal (−9.83 and −9.84 kcal/mol), due to their identical minimum poses (not shown). As expected, the orientations of all the compounds occupy the positive MAMN map adjacent to D147 while also fitting the 4,5-epoxymorphinan core in apolar site A formed by W293 and Y326.

**TABLE 3:** List of the ligands used in SILCS-MC with their experimental Ki(nM), corresponding binding Gibb’s free energy (ΔG, kcal/mol), and the Ligand Grid Free Energy (kcal/mol) scores against the active and inactive conformations of the MOPr and DOPr. Gibb’s free energy error estimates are based on the Ki SEM values converted to a free energy.

Cpd	MOPr				DOPr			
	Ki (±SEM)	ΔG (kcal/mol)	LGFE (kcal/mol)		Ki (±SEM)	ΔG (kcal/mol)	LGFE (kcal/mol)	
			Active	Inactive			Active	Inactive
**7**	19.9 ± 11.7	−10.57 ± 0.28	−9.83	−7.47	55 ± 8	−9.97 ± 0.08	−10.69	−8.12
**8**	23.8 ± 1.2	−10.46 ± 0.03	−7.29	−7.25	47 ± 16	−10.06 ± 0.17	−10.22	−7.76
**9**	39.9 ± 10.5	−10.16 ± 0.14	−9.84	−7.28	121 ± 45	−9.50 ± 0.19	−10.66	−7.86
**10**	11.0 ± 2.4	−10.92 ± 0.12	−7.88	−7.80	37 ± 9	−10.20 ± 0.13	−9.02	−8.63
**11**	22.7 ± 6.0	−10.49 ± 0.14	−8.41	−7.77	37 ± 7	−10.20 ± 0.10	−9.15	−8.73
**12**	0.9 ± 0.3	−12.42 ± 0.17	−9.63	−7.57	10 ± 3	−10.98 ± 0.16	−10.91	−7.97
**13**	74.8 ± 24.1	−9.78 ± 0.17	−9.45	−7.45	56 ± 20	−9.95 ± 0.18	−9.96	−8.09
**14**	1.3 ± 0.8	−12.20 ± 0.29	−10.12	−7.05	36 ± 4	−10.22 ± 0.06	−11.18	−8.48

SILCS calculations on the inactive receptor conformation of MOPr were also conducted. As shown in Figure 5B, the ligands assumed flipped orientations in the inactive MOPr pocket, thus shifting downwards into the apolar pocket and away from D147 and almost 11 Å away from apolar site B (Supplementary Figure S3B). As shown in Figures 4C,D, the apolar, donor and acceptor maps in the inactive MOPr are not abundant in the pocket, when compared to the active conformation. This might be due to a larger pocket size in inactive MOPr caused by different orientations of the side chains of apolar residues lining the pocket. A similar description about SILCS FragMaps and the SILCS-MC poses of the ligands in DOPr is described in the Supporting Material (Supplementary Figure S3).

Overall, from the SILCS calculations, **14** is shown to be a good binder for active MOPr and active and inactive DOPr, suggesting agonism for MOPr and partial agonism to DOPr. The 2-napthyl group of **14** consistently assumed a favorable pose occupying the apolar sites A and B as well as a considerable overlap of its basic amine group with the key site in both MOPr and DOPr. Since the 2-napthyl group has the maximum number of aromatic carbon atoms in the R_2_ group, its overlap with the apolar site B explains its most favorable LGFE scores. On the other hand, 1-napthyl of **13** moved slightly away from the key site to adjust to the longer 1-napthyl R_2_ group, leading to a less favorable contribution by its basic amine nitrogen. Conclusively, the SILCS-MC ligand poses in both MOPr and DOPr receptor pockets indicated that while the basic amine nitrogen of 4,5-epoxymorphinan compounds occupy the message site, the R_2_ groups acts as the “address” groups, thus driving the binding of the ligands into the MOPr/DOPr receptor pockets.

Thus, the SILCS modeling illustrated the differences between the active and inactive conformations of both MOPr and DOPr, indicating the ability of SILCS method to differentiate between active and inactive forms of the same receptors while also differentiating between different receptors belonging to the same receptor family. This ability to differentiate between active and inactive forms of a GPCR was previously used to identify novel orthosteric activators of the β2-adrenergic receptor (Lakkaraju et al., 2015). In the present study, we were able to illustrate the differences inside the active and inactive pockets of MOPr and DOPr, such that SILCS-MC docking predicted the top two compounds **12** and **14** precisely in both MOPr and DOPr receptors. In addition, through the SILCS FragMaps, we were able to locate the important structural differences in the MOPr and DOPr pockets which may be utilized for designing 4,5-epoxymorphinan compounds or other morphine analogs.

## 3 Conclusion

The present results demonstrate, for the first time, that modifications to the *para*-position of the aromatic ring of 7-*E*-benzylidene-substituted 4,5-epoxymorphinans can influence efficacy at opioid receptors. Two compounds **12** and **14**, emerged as high-potency, high-efficacy MOPr agonists and DOPr antagonists with negligible affinity for KOPr in *vitro* tests, and compound **12** produced antinociception *in vivo*. Future SAR studies are underway to determine whether other electron-withdrawing groups are tolerated in this region, and whether modifications to other regions of the aromatic group similarly influence OPr pharmacodynamics. Further, future experiments are planned to delineate the discrepancy between the potency of **12** *in vitro* (2.2 nM) vs *in vivo* (32 mg/kg). Possible explanations include pharmacokinetic effects like rapid metabolism into inactive metabolites, or physicochemical properties like a high topologic polar surface area (TPSA = 115.8) that limits central bioavailability (Wager et al., 2010).

Using SILCS FragMaps, we propose that *p*-aryl substitutions to the benzylidene occupy chemical space that stabilizes the MOPr active state conformation. SILCS further demonstrates the presence of apolar maps, which overlapped with the *p*-aryl substituent groups such as the 2-naphthyl group of **14** and the 4-NO_2_-Ph group of **12**. The SILCS atom-based GFE SAR gives atomistic-level details of the binding affinity of the compounds. Future studies will use these SILCS-defined FragMaps to facilitate the rational design of potent, bifunctional MOPr/DOPr analgesics. The highest affinity/potency compound (**12**) showed improved antinociception activity relative to the parent compound **5** *in vivo* but, unfortunately, **12** also produced similar antinociceptive tolerance to the classical MOPr agonist, morphine. Nonetheless, these derivatives are important tools to better understand OPr binding and efficacy on a molecular level and represent new MOPr/DOPr leads for future SAR studies.

## 4 Experimental

### 4.1 Chemistry

Solvents and reagents of commercial-grade were purchased from Millipore-Sigma and were used without additional purification. All benchtop reactions were run in oven-dried flasks. Microwave reactions were conducted using an Initiator^®^ (Biotage, Inc., Uppsala, Sweden) microwave apparatus using their standard reaction vessels. Compounds were purified by automated flash column chromatography using an Isolera^®^ apparatus (Biotage, Inc). ^1^H and ^13^C NMR spectra were obtained using a 500 MHz Varian NMR. Melting points were determined in open capillary tubes using a Mel-Temp melting point apparatus. TLC was performed on silica gel 60 GF plates (Analtech, Inc., Newark, DE). Purity was determined by HPLC using an UltiMate 3000 HPLC system using a Kinetex^®^ 5 μM EVO C18 100 Å LC column, 150 × 4.6 mm (Phenomenex, Torrance, CA). Oxymorphone was purchased from Mallinckrodt, Inc (St. Louis, MO). Full details of the synthesis and characterization of compounds is found in Supplementary Material S1.

### 4.2 *In vitro* pharmacology

**Cell lines, cell culture, and drug preparation.** The hDOPr-CHO cell line was a generous gift from Dr. Larry Toll at the Torrey Pines Institute. The hMOPr- and hKOPr-CHO cell lines were generous gifts from John M. Streicher at the University of Arizona. All cells were cultured in 50:50 DMEM/F12 media with 10% heat-inactivated FBS and 1X penicillin/streptomycin supplement (all Gibco brand) in a 37°C humidified incubator with 5% CO_2_ atmosphere. Propagation cultures were further maintained with 500 μg/mL G418. Cultures were propagated for no more than 30 passages before discarding. Cell pellets for experiments were prepared by growth in 15 cm^2^ plates, harvested with 5 mM EDTA in 50 mM Tris HCl pH 7.4. Cell pellets were resuspended in 50 mM Tris HCl pH 7.4 and homogenized with a tissue grinder for 15–30 s on ice. Crude membranes were spun down at 15,000 g for 30 min. This process was repeated, and membrane preparations were stored at −80°C prior to use.

For *in vitro* experiments, stock drugs were dissolved in DMSO to 10 mM and stored at −20°C. The day of the assay, drug preparations were aliquoted in the assay buffer indicated below to the indicated concentrations, maintaining a constant DMSO concentration of 0.4% in all wells.

**Competition radioligand binding.** hMOPr-, hDOPr- or hKOPr-CHO membrane preparations were diluted to 10–20 µg/reaction with a fixed concentration of ^3^H-diprenorphine and varying concentrations of competitor ligand, similar to previously described experiments (Olson et al., 2018; Olson et al., 2019; Hillhouse et al., 2021; Keresztes et al., 2021). Briefly, these reactions were miniaturized to a 400 μL volume in 96 well plates and incubated at RT for 1 h at DOPr-, KOPr- and MOPr-CHO membrane preparations. The reactions were terminated by rapid filtration through 96-well format GF/B filter plates (PerkinElmer) and washed with cold 25 mM Tris HCl pH 7.4 buffer, dried, and added ECOLUME^TM^ scintillation cocktail from MP Biomedicals (Santa Cruz, CA, United States). The plates were read in a MicroBeta2 96-well format 6 detector scintillation counter (PerkinElmer). K_I_ values were calculated using the IC_50_ of each competitor ligand and the previously established K_D_ of ^3^H-diprenorphine in each cell line using a competition binding model (GraphPad Prism 7.0).

**[** ^**35**^ **S]GTPγS coupling.** Briefly, hDOPr, hKOPr, and hMOPr-CHO membranes were combined with drug/control concentration curves, ∼50 p.m. ^35^S-GTPγS (Perkin Elmer), and 10–15 µg/reaction in GTPγS assay buffer (50 mM Tris HCl pH 7.4, 125 mM NaCl, 5 mM MgCl_2_, 1 mM EDTA and 30 µM GDP) as previously reported (Schiller et al., 1999; Ananthan, 2006). The 200 μL reaction was incubated at 30°C for 60 min in 96 well plates, then collected and measured as for the binding experiments. EC_50_ and E_MAX_ values were calculated using the three-variable log (agonist) vs response curve in GraphPad Prism 7.0. IC_50_ and I_MAX_ values were calculated using the three-variable log (inhibitor) vs response curve in GraphPad Prism 7.0.

**βarrestin2 recruitment.** βarrestin2 recruitment in CHO cells expressing MOPr was measured using the DiscoverRx PathHunter^®^ assay kit (DiscoveRx, Fremont, CA) according to the manufacturer’s protocol. Assays were performed on at least three separate occasions in triplicate. Luminescence was read using a Synergy 2 plate reader (BioTek, Winooski, VT).

### 4.3 *In vivo* pharmacology

**Drug preparation.** All compounds were administered *i. p.* Dissolved in 1% 0.1 N HCl, 5 %DMSO, 94% H_2_O vehicle for most experiments or s. c. injection dissolved in a vehicle of 2.5% Tween20, 2.5% DMSO, 95% sterile H_2_O.

**Animals.** All animal care and experimental protocols were performed in accordance with the United States National Research Council’s Guide for the Care and Use of Laboratory Animals and approved by the University of Michigan Institutional Animal Care and Use Committee.

Male and female C57BL/6 MOPr knock out (KO) mice (B6.129S2-*Oprm1* ^*tm1Kff*^/J stock number 007559; Jackson Laboratory), or C57BL/6 wild-type mice (stock number 000664; Jackson Laboratory) weighing between 20 and 30 g at 8–16 weeks old, were used for the 50 C WWTW experiments. 129S1/SvImJ mice (Invigo) were used for the 55 C WWTW acute antinociception experiments. All KO animals were bred in-house from heterozygous breeders. Mice were group housed with a up to five animals per cage with free access to food and water. Animals housing was in rooms with a temperature between 20°C and 26°C and with 30%–70% humidity with a 12 h light/dark cycle with lights on at 07:00 h; experiments proceeding during the light cycle.

**Acute antinociception: 50°C warm-water tail withdrawal test in C57BL/6 mice.** In C57BL6 WT mice, the 50°C warm-water tail withdrawal (WWTW) test was used to evaluate antinociception upon *i. p.* Administration of vehicle (1% 0.1 N HCl, 5 %DMSO, 94% H_2_O), morphine, the parent compound **5**, or the lead **12** as described in Figure 2A. Briefly, the mouse’s distal tip of the tail (∼1/3) was placed in a 50°C warm-water bath and the latency for the mouse to flick its tail was measured. A maximum cutoff time of 20 s was implemented to prevent tissue damage. Tail withdrawal latencies were measured at the indicated times or 30 min after drug injection using a cumulative dosing procedure as previously reported (Healy et al., 2013). Drug-stimulated antinociception was expressed as a percentage of maximum possible effect (% MPE), where % MPE = (drug latency—baseline latency)/(cutoff latency—baseline latency) × 100. The acute antinociception results were analyzed with a one-way ANOVA followed by a *post hoc* Dunnett test comparing the drug(s) to vehicle.

**Chronic administration and antinociceptive tolerance.** To investigate antinociceptive tolerance, either C57BL/6 WT or MOR KO mice were administered (*i.p.*) drug 2-3 times a day as indicated (Figure 3). On day 1 at 1 p.m., mice were weighed, injected (*i.p.*) with **12**, **5**, morphine, or vehicle (1% 0.1 N HCl, 5% DMSO, 94% H_2_O) 30 min before testing in the WWTW assay. Mice administered with **12** were injected with 0.5 mL 0.9% saline (*s.c.*) twice daily. Mice were injected again at 7 p.m. on day 1, but neither weighed nor tested for antinociception. On days 2–4, mice were injected *i. p* with drug doses indicated at 7 a.m., 1 p.m., and 7 p.m. Mice were again weighed and tested for antinociception at 1 p.m. For the naloxone experiment, naloxone (10 mg/kg, *i. p.*) was administered 30 min before administration of 32 mg/kg **12**. A two-way analysis of variance (ANOVA) was conducted for all tolerance assays. The significance threshold was *p* < 0.05, and a Tukey *post hoc* test followed all significant ANOVAs.

### 4.4 Computation

**System preparation for SILCS.** SILCS oscillating chemical potential Grand Canonical Monte Carlo/Molecular Dynamics (GCMC/MD) simulations (Lakkaraju et al., 2014) for the active and inactive forms of MOPr and DOPr were initiated from X-ray crystal structures of the mouse receptor with PDB identification codes 5C1M (active MOPr) ^57^and 4DKL (inactive MOPr) (Manglik et al., 2012) and 4EJ4 (inactive DOPr). To study the active form of DOPr, a homology model was built using PDB ID 6PT3 (Claff et al., 2019) as the template and equilibrated for 500 ns with restraints of 5 kcal/mol on backbone non-hydrogen atoms. Despite the availability of 6PT3—a human active DOPr—the homology model was preferred to avoid structural changes that the point mutations may have caused to 6PT3. The agonist and G protein mimetic nanobody in 5C1M and the antagonist and T4-lysozyme (T4L) inserted in intracellular loop 3 (ICL3) of 4DKL, along with other ligands except for the co-crystallized cholesterol molecule, were removed. The ICL3 loop (residues 263–282) was then modeled into the 4DKL structure along with missing residues 51 to 64 of the N-terminal of the protein using Modeller (Version 9.11) (Sali and Blundell, 1993; Sali and Overington, 1994; Fiser et al., 2000; Sánchez and Sali, 2000), with the top-scoring model out of 500 generated conformations chosen for system preparation. Both protein structures were then aligned along the *Z*-axis for membrane insertion using the Orientations of Proteins in Membranes (OPM) webserver (Lomize et al., 2012). Both structures were inserted into POPC-Cholesterol (9:1) lipid bilayer systems using Membrane Builder (Bilayer Builder) of CHARMM-GUI (Jo et al., 2007; Jo et al., 2008; Jo et al., 2009; Wu et al., 2014; Lee et al., 2016; Lee et al., 2019). Residue YCM57, a modified cysteine residue in 5C1M, was replaced by a standard cysteine. GROMACS was then used for further system equilibration. To obtain a stable conformation of the rebuilt N-terminal loop in the inactive crystal structure (4dkl), a 100 ns molecular dynamics (MD) simulation was undertaken with GROMACS 2018.1 (Hess et al., 2008), with backbone non-hydrogen atom restraints of 5 kcal/mol. The resulting RMSD of the protein backbone without the extracellular and intracellular loop regions was 1.5 Å. To model the active DOPr structure, 6PT3 was used as the template. Monomer A was extracted from the dimeric crystal structure, followed by system preparation in Molecular Operating Environment (MOE), which changed the point mutations to their wildtype amino acid residues. This model was then further processed to build the missing residues using Modeller, as described above, and then equilibrated for −160 ns using backbone non-hydrogen atom restraints of 5 kcal/mol in GROMACS. This equilibrated system was then used as a template for developing the homology model of the active mouse DOP receptor. The sequence for the mouse structure was obtained from UniProt (Consortium, 2019) (UniProt ID P32300) and was modelled using Modeller, where the top model of 100 different conformations was chosen for further system preparation. The system was prepared in CHARMM-GUI, as with the other three receptors, and was equilibrated with backbone non-hydrogen atom restraints of 5 kcal/mol for −500 ns The equilibrated system was then used for the SILCS simulations.

**SILCS simulations.** The systems for the SILCS simulations were prepared by solvating with TIP3P water (55 M) and standard SILCS solutes (0.25 M) with SILCS-membrane module of the SILCS MolCal program version 2022.1 (SilcsBio, LLC) (Yu et al., 2015; Raman et al., 2017; Ustach et al., 2019). The solutes used for the study included benzene, propane, acetaldehyde, methanol, formaldehyde, imidazole, acetate, and methylammonium. Force field parameters for the protein were CHARMM36 m (Best et al., 2012; Huang et al., 2017) with CGenFF (Vanommeslaeghe et al., 2010; Vanommeslaeghe et al., 2012; Vanommeslaeghe and MacKerell, 2012) used for the solutes and studied ligands. All ligands contained a formal charge of +1 on the basic amine Nitrogen, with respective force field parameters from CGenFF. The simulations involve the hybrid GCMC/MD approach as previously described in detail (Lakkaraju et al., 2014). After the GCMC-MD simulation was finished, the functional group occupancy maps, called FragMaps, were generated as previously described (Ustach et al., 2019).

**Computation of binding affinity of ligands.** Estimating binding affinities was performed using SILCS-MC (Lakkaraju et al., 2014; Ustach et al., 2019). SILCS-MC involves Monte Carlo conformational sampling of the ligands in the field of the SILCS FragMaps, where the Metropolis criteria are based on the Ligand Grid Free Energy (LGFE) scores plus the CGenFF intramolecular energy based on a distance-dependent dielectric constant. LGFEs were calculated as the overlap of the classified atoms in the ligands with the respective FragMaps from which the Grid Free Energy (GFE) for each classified atom was obtained. GFE of each atom of a molecule sampled in SILCS-MC is calculated on the basis of per atom overlap with the respective maps representing the individual chemical functionalities (Raman et al., 2011; Raman et al., 2013; Lakkaraju et al., 2014; Yu et al., 2014; Faller et al., 2015; Yu et al., 2015; Raman et al., 2017; Ustach et al., 2019). The atomic GFE scores are summed to yield the final LGFE scores. While MC sampling is based on the LGFE plus CGenFF intramolecular energies, final scores for the ligands are based on the LGFE scores alone.

Preparation of the BOM ligands for SILCS-MC sampling involved alignment with BU72, the cocrystal agonist from 5C1M (active model), using the “flexible alignment” module of Molecular Operating Environment (MOE) (Molecular Operating Environment, 2019). The ligands were sampled using the aligned poses as the initial orientation over a radius of 10 Å from the center of mass of the initial orientation. Ligands were initially energy minimized for 10,000 steps based on the CGenFF energy function prior to SILCS-MC. SILCS-MC was comprised of MC and simulated annealing sampling. MC involved 10,000 moves of maximum molecular translation of 1.0 Å, molecular rotation of 180° and dihedral rotation of 180° followed by 40,000 steps of simulated annealing where each move was comprised of a maximum of 0.2 Å molecular translation, 9.0° molecular rotation and 9.0° dihedral rotation. Each ligand was sampled for a total of 250 such cycles, similar to the “exhaustive” SILCS-MC protocol described in reference (Ustach et al., 2019). The lowest LGFE conformation was used for SAR analysis. The structures of the 7-*E*-benzylideneoxymorphone (BOM) ligands used for this study are shown in Scheme 1.

## Data Availability

The datasets presented in this study can be found in online repositories. The names of the repository/repositories and accession number(s) can be found in the article/Supplementary Material.
