# Supplementary material for: Nitro-benzylideneoxymorphone, a bifunctional mu and delta opioid receptor ligand with high mu opioid receptor efficacy
Source: Front Pharmacol. 2023 Jul 3;14:1230053. doi: 10.3389/fphar.2023.1230053 (PMC10352325; doi:10.3389/fphar.2023.1230053)
Supplement: Supplementary file 1 [file DataSheet1.PDF]

Supplementary Material

Nitro-Benzylideneoxymorphone, a Bifunctional Mu and Delta Opioid Receptor Ligand with High Mu Opioid Receptor Efficacy

Keith M. Olson[1†], Andrea L. Devereaux[2†], Payal Chatterjee[3†], Savanah L. Saldaña-Shumaker[2], Amanda Shafer[1], Adam Plotkin[2], Ram Kandasamy[1,4], Alexander D. MacKerell Jr.[3], John R. Traynor[1,5], Christopher W. Cunningham[2*]

*** Correspondence:** Corresponding Author: chris.cunningham@cuw.edu

1 Synthesis and characterization of compounds

Chemistry. Solvents and reagents of commercial-grade were purchased from Sigma-Aldrich and were used without additional purification. All benchtop reactions were run in oven-dried flasks. Microwave reactions were conducted using an Initiator® (Biotage, Inc., Uppsala, Sweden) microwave apparatus using their standard reaction vessels. Compounds were purified by automated flash column chromatography using an Isolera® apparatus (Biotage, Inc). ^{1}H and ^{13}C NMR spectra were obtained using a 500 MHz Varian NMR. Melting points were determined in open capillary tubes using a Mel-Temp melting point apparatus. TLC was performed on silica gel 60 GF plates (Analtech, Inc., Newark, DE). Oxymorphone was purchased from Mallinckrodt, Inc (St. Louis, MO).

High-Performance Liquid Chromatography (HPLC). Compound purity was determined using an UltiMate 3000 HPLC system using a Kinetex® 5 μM EVO C18 100 Å LC column, 150x4.6 mm (Phenomenex, Torrance, CA). Mobile phases used were 0.1% formic acid in Milli-Q water (A) and 0.1% formic acid in acetonitrile (B). An isocratic method was used over 40 minutes: 95% A and 5% B from 0-25 minutes; 100% B from 25-31 minutes; 95% A and 5% B from 31-40 minutes. The flow rate was 0.5 mL/min from 0-26 minutes, 1.0 mL/min from 26-29 minutes, and 0.5 mL/min from 39-40 min. The column temperature was 25°C. Detection was obtained at 220.0, 254.0, 280.0, and 300.0 nm. The reference bandwidths were 2 nm and 10 nm, respectively, for all UV-Vis levels. All compounds were confirmed to have purity > 95% prior to testing.

Method A: Sealed Tube. Methanol or ethanol (0.1M) was added to oxymorphone hydrochloride (1 equiv.) in a sealed tube, followed by aldehyde (6.0 equiv.) and piperidine (2 equiv). The tube was sealed, and the mixture was heated at 120°C for 24 hr. After cooling to RT, the mixture was partitioned between H_2O and $CHCl_3$ and made acidic (pH 1) with HCl. The aqueous phase was washed three times with $CHCl_3$. The organic phase was removed, the aqueous phase made basic (NH_4OH), and extracted 3x into $CHCl_3$. The combined organic phases were dried over Na_2SO_4 and solvent removed *in vacuo*.

Method B: Microwave. To a microwave vial of oxymorphone hydrochloride (1.0 equiv.) in EtOH (0.1M) was added aldehyde (6 equiv.) and piperidine (2 equiv). The vial was sealed and subjected to microwave irradiation at 160°C for 1 hr. During the reaction, pressure readings reached between 10-12 bar. After cooling to RT, the mixture was partitioned between H_2O and $CHCl_3$ and made acidic (pH 1) with HCl. The aqueous phase was washed three times with $CHCl_3$. The organic

phase was removed, the aqueous phase made basic (NH_4OH), and extracted 3x into $CHCl_3$. The combined organic phases were dried over Na_2SO_4 and solvent removed *in vacuo*.

17-methyl-3,14β-dihydroxy-4,5α-epoxy-7-(*E*-phenylmethylene)-morphinan-6-one (BOM, 5). Method A. Oxymorphone.HCl (101.1 mg, 0.299 mmol), benzaldehyde (0.18 mL, 1.776 mmol), piperidine (0.060 mL, 0.6 mmol) were combined in MeOH (3 mL) and reacted according to General Method A. Crude product was purified by flash column chromatography (EtOAc) to afford 25.9 mg (0.067 mmol, 22% yield) of **5** as a yellow solid.

Method B. Oxymorphone.HCl (50.7 mg, 0.166 mmol), benzaldehyde (0.09 mL, 1.089 mmol), piperidine (0.030 mL, 0.3 mmol) were combined in EtOH (1.5 mL) and reacted according to General Method B. Crude product was purified by flash column chromatography (EtOAc) to afford 32.1 mg (0.082 mmol, 50% yield) of **5** as a yellow solid.

^{1}H NMR (500 MHz, $CDCl_3$) δ 7.70 (s, 1H), 7.37 (m, 5H), 6.78 (d, J=8, 1H), 6.69 (d, J=6.5, 1H), 4.73 (s, 1H), 4.16 (q, J=6.5, 1H), 3.28 (d, J=19, 1H), 3.04 (d, J=15, 1H), 2.93 (d, J-6.5, 1H), 2.71 (d, J=7, 1H), 2.67 (d, J=6.5, 1H), 2.60 (d, J=6, 1H), 2.40 (m, 7H), 2.09 (s, 3H), 1.68 (d, J-10.5, 1H), 1.30 (t, J=7, 2H). ^{13}C NMR (500 MHz, $CDCl_3$) δ 221.4, 160.4, 141.1, 139.7, 138.3, 130.8, 129.5, 129.0, 127.7, 124.7, 120.7, 119.4, 118.1, 116.8, 110.0, 110.0, 109.9, 104.5, 89.5, 63.7, 47.4. M.p. 110-120°C. HPLC 97.7%. MS (ESI) calculated for $C_{24}H_{23}NO_4$, 389.4; observed *m/z* 389.5 ($M+H^+$).

17-methyl-3,14β-dihydroxy-4,5α-epoxy-7-(*E*-4′-fluorophenylmethylene)-morphinan-6-one (7). Oxymorphone.HCl (101.1 mg, 0.299 mmol), 4-fluorobenzaldehyde (0.19 mL, 1.776 mmol), piperidine (0.060 mL, 0.6 mmol) were combined in EtOH (3 mL) and reacted according to Method A. Solution turned bright yellow on the addition of piperidine, which turned orange after heating for several hours. The crude product was purified by flash column chromatography (EtOAc) to afford 60 mg (0.147 mmol, 50%) of **7** as a yellow solid.

^{1}H NMR (500 MHz, $CDCl_3$) δ 7.55 (s, 1H), 7.28 (d, 2H, J = 8.5 Hz), 7.01 (d, 2H, J = 8.5 Hz), 6.71 (d, 1H, J = 8.5 Hz), 6.62 (d, 1H, J = 8.5 Hz), 4.64 (s, 1H), 3.22 (d, 1H, J = 18.5 Hz), 2.91 (d, 1H, J = 22 Hz), 2.88 (d, 1H, J = 12.5 Hz), 2.63 (dd, 1H, J = 18.5, 6.5 Hz), 2.37 (s, 3H), 2.20-2.40 (m, 4H), 1.61 (d, 1H, J = 9.5 Hz). ^{13}C NMR (500 MHz, $CDCl_3$) δ 143.7, 139.2, 138.2, 132.1, 132.0, 132.0, 131.2, 129.7, 124.6, 120.1, 117.4, 115.6, 115.4, 110.0, 109.9, 90.0, 90.0, 70.4, 64.3, 47.2, 44.9, 42.9, 33.5, 31.6, 22.2, 22.2. M.p. 240°C (dec). HPLC 96.8%. MS (ESI) calculated for $C_{24}H_{22}FNO_4$, 407.4; observed *m/z* 407.4 ($M+H^+$).

17-methyl-3,14β-dihydroxy-4,5α-epoxy-7-(*E*-4′-chlorophenylmethylene)-morphinan-6-one (8). Oxymorphone.HCl (100.8 mg, 0.298 mmol), 4-chlorobenzaldehyde (249.8 mg, 1.776 mmol), piperidine (0.060 mL, 0.6 mmol) were combined in EtOH (3 mL) and reacted according to Method A. Solution turned yellow after heating for 1 hr. The crude product was purified by flash column chromatography (EtOAc) to afford 42.1 mg (0.099 mmol, 33%) of **8** as a white solid.

^{1}H NMR (500 MHz, $CDCl_3$) δ 7.57 (s, 1H), 7.33 (d, 2H, J = 8.5 Hz), 7.27 (d, 2H, J = 8.5 Hz), 6.75 (d, 1H, J = 8 Hz), 6.66 (d, 1H, J = 8 Hz), 4.68 (s, 1H), 3.25 (d, 1H, J = 18 Hz), 2.93 (d, 1H, J = 15 Hz), 2.89 (d, 1H, J = 6 Hz), 2.65 (dd, 1H, J = 18.5, 6 Hz), 2.40 (s, 3H), 2.32-2.38 (m, 4H), 1.65 (d, 1H, J = 10.5 Hz). ^{13}C NMR (500 MHz, $CDCl_3$) δ 197.9, 138.2, 132.0, 131.5, 130.7, 130.2, 129.4, 128.8, 128.8, 128.0, 120.7, 119.5, 118.1, 110.1, 110.0, 110.0, 109.9, 109.9, 109.9, 89.5, 63.7, 33.5. M.p. 240°C (dec). HPLC 97.4%. MS (ESI) calculated for $C_{24}H_{22}ClNO_4$, 423.8; observed *m/z* 423.9 ($M+H^+$).

17-methyl-3,14β-dihydroxy-4,5α-epoxy-7-(*E*-4′-bromophenylmethylene)-morphinan-6-one (9). Oxymorphone.HCl (49.9 mg, 0.148 mmol), 4-bromobenzaldehyde (164.4 mg, 0.888 mmol), piperidine (0.030 mL, 0.3 mmol) were combined in EtOH (1.5 mL) and reacted according to Method B. Solution turned yellow during the reaction. The crude product was purified by flash column chromatography (90:10 EtOAc:hexanes) to afford 32.1 mg (0.069 mmol, 46% yield) of **9** as a white solid.

^{1}H NMR (500 MHz, $CDCl_3$) δ 7.54 (s, 1H), 7.48 (d, 2H, J = 8 Hz), 7.20 (d, 2H, J = 8 Hz), 6.74 (d, 1H, J = 8 Hz), 6.65 (d, 1H, J = 8 Hz), 4.68 (s, 1H), 3.24 (d, 1H, J = 18.5 Hz), 2.92 (d, 1H, J = 15.5 Hz), 2.88 (d, 1H, J = 6 Hz), 2.64 (dd, 1H, J = 18.5, 6.5 Hz), 2.49 (d, 1H, J = 7.5 Hz), 2.40 (s, 3H), 2.30-2.36 (m, 3H), 1.65 (d, 1H, J = 10 Hz). ^{13}C NMR (500 MHz, $CDCl_3$) δ 138.3, 134.0, 132.9, 132.3, 132.3, 132.2, 132.1, 131.0, 130.9, 124.6, 120.8, 119.5, 118.1, 116.9, 110.0, 109.9, 109.9, 109.9, 89.4, 70.5, 43.4, 23.2, 22.2. M.p. 220°C (dec). HPLC 97.3%. MS (ESI) calculated for $C_{24}H_{22}BrNO_4$, 468.3; observed *m/z* 468.4 ($M+H^+$).

17-methyl-3,14β-dihydroxy-4,5α-epoxy-7-(*E*-4′-methoxyphenylmethylene)-morphinan-6-one (10). Oxymorphone.HCl (50 mg, 0.148 mmol), *p*-anisaldehyde (0.108 mL, 0.888 mmol), piperidine (0.030 mL, 0.3 mmol) were combined in EtOH (15 mL) and reacted according to Method B. Crude product was purified by flash column chromatography (90:10 EtOAc:hexanes) to afford 12 mg (0.029 mmol, 20% yield) of **10** as a yellow foam.

^{1}H NMR (500 MHz, $CDCl_3$) δ 7.64 (s, 1H), 7.31 (d, 2H, J = 8.5 Hz), 6.88 (d, 2H, J = 8.5 Hz), 6.74 (d, 1H, J = 8 Hz), 6.67 (d, 1H, J = 8 Hz), 4.67 (s, 1H), 3.25 (d, 1H, J = 18.5 Hz), 3.00 (d, 1H, J = 15.5 Hz), 2.90 (d, 1H, J = 6.5 Hz), 2.67 (dd, 1H, J = 18.5, 6.5 Hz), 2.49 (d, 1H, J = 6.5 Hz), 2.39 (s, 3H), 2.05-2.36 (m, 3H), 1.65 (d, 1H, J = 9.5 Hz). ^{13}C NMR (500 MHz, $CDCl_3$) δ 143.7, 138.2, 132.9, 131.6, 130.0, 120.6, 119.3, 118.0, 116.8, 114.5, 114.5, 113.3, 110.0, 109.9, 90.6, 89.5, 55.9, 54.7, 44.9, 41.5, 33.8. M.p. 109-111°C (dec). HPLC 95.6%. MS (ESI) calculated for $C_{25}H_{25}NO_5$, 419.5; observed *m/z* 419.7 ($M+H^+$).

17-methyl-3,14β-dihydroxy-4,5α-epoxy-7-(*E*-4′-hydroxyphenylmethylene)-morphinan-6-one (11). Oxymorphone.HCl (100.8 mg, 0.298 mmol), 4-hydroxybenzaldehyde (217.2 mg, 1.776 mmol), piperidine (0.060 mL, 0.6 mmol) were combined in MeOH (3 mL) and reacted according to Method A with a slight modification on workup. The solution turned bright yellow with the addition of piperidine. After cooling to RT, the mixture was partitioned between H_2O and $CHCl_3$ and made acidic (pH 1) with HCl. The aqueous phase was washed 3x with $CHCl_3$. The organic phase was removed, the aqueous phase made basic ($NaHCO_3$), and extracted 3x into $CHCl_3$. The crude product was purified by flash column chromatography (99:1 EtOAc:CH_2Cl_2) to afford 32.1 mg (0.079 mmol, 27%) of **11** as a yellow solid.

^{1}H NMR (500 MHz, $CDCl_3$) δ 7.62 (s, 1H), 7.24 (d, 2H, J = 8.5 Hz), 6.81 (d, 2H, J = 8.5 Hz), 6.74 (d, 1H, J = 8.5 Hz), 6.65 (d, 1H, J = 8.5 Hz), 4.67 (s, 1H), 3.25 (d, 1H, J = 18 Hz), 3.00 (d, 1H, J = 15.5 Hz), 2.91 (d, 1H, J = 6 Hz), 2.68 (dd, 1H, J = 18.5, 6.5 Hz), 2.49 (d, 1H, J = 5.5 Hz), 2.39 (s, 3H), 2.17-2.36 (m, 3H), 1.64 (d, 1H, J = 10 Hz). ^{13}C NMR (500 MHz, $CDCl_3$) δ 166.8, 138.5, 134.6, 132.6, 131.8, 128.9, 128.8, 128.3, 127.7, 127.5, 127.3, 127.1, 126.7, 123.6, 109.9, 60.8, 60.1, 52.7, 49.2, 32.7. M.p. 190°C (dec). HPLC 95.7%. MS (ESI) calculated for $C_{24}H_{23}NO_5$, 405.4; observed *m/z* 405.4 ($M+H^+$).

17-methyl-3,14β-dihydroxy-4,5α-epoxy-7-(*E*-4′-nitrophenylmethylene)-morphinan-6-one (12). Oxymorphone.HCl (50.4 mg, 0.149 mmol), 4-nitrobenzaldehyde (134.1 mg, 0.888 mmol),

piperidine (0.030 mL, 0.3 mmol) were combined in EtOH (1.5 mL) and reacted according to Method B. Solution turned light brown/maroon during the reaction. The crude product was purified by flash column chromatography (EtOAc) to afford 23.7 mg (0.543 mmol, 36% yield) of **12** as a yellow solid.

^{1}H NMR (500 MHz, $CDCl_3$) δ 8.24 (d, 2H, J = 8 Hz), 7.59 (s, 1H), 7.54 (d, 2H, J = 8 Hz), 6.78 (d, 1H, J = 8 Hz), 6.69 (d, 1H, J = 8 Hz), 4.72 (s, 1H), 3.27 (d, 1H, J = 18.5 Hz), 2.91 (d, 1H, J = 15 Hz), 2.91 (d, 1H, J = 6.5 Hz), 2.64 (dd, 1H, J = 18.5, 6.5 Hz), 2.53-2.55 (m, 1H), 2.46 (s, 3H), 2.33-2.43 (m, 3H), 1.69 (d, 1H, J = 12 Hz). ^{13}C NMR (500 MHz, $CDCl_3$) δ 199.5, 144.1, 140.0, 135.1, 135.1, 132.7, 130.1, 129.7, 129.3, 128.8, 128.3, 120.1, 118.6, 109.9, 89.5, 70.5, 64.3, 47.1, 45.8, 15.0, 42.9, 33.5, 31.4, 25.5, 24.2, 22.1. M.p. 155°C (dec). HPLC 97.3%. MS (ESI) calculated for $C_{24}H_{22}N_2O_6$, 434.4; observed *m/z* 434.5 ($M+H^+$).

17-methyl-3,14β-dihydroxy-4,5α-epoxy-7-(*E*-naphthalen-1-ylmethylene)-morphinan-6-one (13). Oxymorphone.HCl (50.1 mg, 0.148 mmol), 1-naphthaldehyde (0.121 mL, 0.888 mmol), piperidine (0.030 mL, 0.3 mmol) were combined in EtOH (1.5 mL) and reacted according to Method B. Solution turned brown during the reaction. Crude product was purified by flash column chromatography (90:10 EtOAc:hexanes) to afford 7.6 mg (0.017 mmol, 12% yield) of **18** as a yellow solid.

^{1}H NMR (500 MHz, $CDCl_3$) δ 8.13 (s, 1H), 7.92 (d, 1H, J = 9.5 Hz), 7.83-7.86 (m, 2H), 7.49-7.51 (m, 2H), 7.43-7.46 (m, 1H), 7.37 (d, 1H, J = 7 Hz), 6.77 (d, 1H, J = 8 Hz), 6.65 (d, 1H, J = 8 Hz), 4.76 (s, 1H), 3.19 (d, 1H, J = 18.5 Hz), 2.86 (d, 1H, J = 15 Hz), 2.80 (d, 1H, J = 6 Hz), 2.57 (dd, 1H, J = 18.5, 6 Hz), 2.49 (dd, 1H, J = 11, 4.5 Hz), 2.36 (s, 3H), 2.28-2.42 (m, 3H), 1.66 (d, 1H, J = 12.5 Hz). ^{13}C NMR (500 MHz, $CDCl_3$) δ 198.5, 138.6, 134.2, 133.4, 131.9, 129.7, 129.2, 128.4, 126.9, 126.5, 126.2, 124.9, 124.8, 120.0, 117.3, 109.9, 109.9, 70.5, 64.2, 47.5, 44.9, 42.8, 33.5, 31.1. M.p. 110-112°C. HPLC 96.0%. MS (ESI) calculated for $C_{28}H_{25}NO_4$ 439.5; observed m/z 439.7 ($M+H^+$).

17-methyl-3,14β-dihydroxy-4,5α-epoxy-7-(*E*-naphthalen-2-ylmethylene)-morphinan-6-one (14). Oxymorphone.HCl (50.0 mg, 0.148 mmol), 2-naphthaldehyde (138.7 mg, 0.888 mmol), piperidine (0.030 mL, 0.3 mmol) were combined in EtOH (1.5 mL) and reacted according to Method B. Solution turned brown during the reaction, then yellow during workup. The crude product was purified by flash column chromatography (90:10 EtOAc:hexanes) to afford 36.2 mg (0.082 mmol, 56% yield) of **14** as a yellow solid.

^{1}H NMR (500 MHz, $CDCl_3$) δ 7.70-7.90 (m, 6H), 7.48-7.49 (m, 2H), 7.44 (d, 1H, J = 8 Hz), 6.76 (d, 1H, J = 8 Hz), 6.67 (d, 1H, J = 8.5 Hz), 4.71 (s, 1H), 3.24 (d, 1H, J = 18.5 Hz), 3.10 (d, 1H, J = 15 Hz), 2.89 (d, 1H, J = 6 Hz), 2.67 (dd, 1H, J = 18.5, 6 Hz), 2.39 (s, 3H), 2.04-2.49 (m, 3 H), 1.65 (d, 1 H, J = 10.5 Hz). ^{13}C NMR (500 MHz, $CDCl_3$) δ 198.3, 192.3, 171.2, 143.9, 140.5, 138.5, 136.4, 134.5, 134.1, 133.1, 132.9, 132.7, 132.6, 132.5, 130.1, 129.7, 129.5, 129.1, 129.0, 128.4, 128.0, 127.9, 127.6, 127.2, 127.0, 126.9, 126.4, 124.4, 122.7, 120.0, 117.7, 109.9, 109.9, 89.9, 70.5, 64.2, 60.4, 47.2, 44.9, 42.8, 33.7, 31.5, 22.2, 21.0, 14.2. M.p. 103-107°C. HPLC 95.9%. LRMS (ESI) calculated for $C_{28}H_{25}NO_4$, 439.5; observed *m/z* 439.6 ($M+H^+$).

17-methyl-3,14β-dihydroxy-4,5α-epoxy-7-(*E*-cyclohexylmethylene)-morphinan-6-one (15). Oxymorphone.HCl (50.2 mg, 0.149 mmol), cyclohexanecarboxaldehyde (0.108 mL, 0.888 mmol), piperidine (0.030 mL, 0.3 mmol) were combined in EtOH (1.5 mL) and reacted according to Method B. Solution turned orange/brown during the reaction. Crude product was purified by flash

column chromatography (90:10 EtOAc:hexanes) to afford 17.8 mg (0.045 mmol, 30% yield) of **17** as a yellow/orange solid.

^{1}H NMR (500 MHz, $CDCl_3$) δ 6.71 (d, 1H, J = 8.5 Hz), 6.61 (d, 1H, J = 8.5 Hz), 6.55 (dd, 1H, J = 2.9, 9.5 Hz), 4.57 (s, 1H), 3.21 (d, 1H, J = 19 Hz), 2.90 (d, 1H, J = 6.5 Hz), 2.63 (d, 1H, J = 15 Hz), 2.60 (dd, 1H, J = 6.5, 18.5 Hz), 2.45-2.49 (m, 1H), 2.40 (s, 3H), 2.33-2.39 (m, 1H), 2.28-2.33 (m, 2H), 2.09 (dd, 1H, J = 2.5, 15.5 Hz), 1.59-1.73 (m, 3H), 1.00-1.40 (m, 7H). ^{13}C NMR (500 MHz, $CDCl_3$) δ 226.1, 157.7, 156.2, 143.7, 117.6, 115.9, 112.3, 112.3, 110.0, 90.7, 67.0, 66.9, 60.7, 60.0, 37.7, 35.6, 33.0, 32.7, 30.9, 26.7, 26.6, 22.8. M.p. 200°C (dec). HPLC 97.7%. MS (ESI) calculated for $C_{24}H_{29}NO_4$, 395.5; observed *m/z* 395.6 ($M+H^+$).

2 DOPR arrestin recruitment assay

Figure S1. DOPr arrestin recruitment assay. Various concentrations of drugs were tested for agonist activity using DiscoveRx βarrestin2 recruitment assay. E_{max}% calculated as percent maximal standard agonist, SNC80, at DOPr.

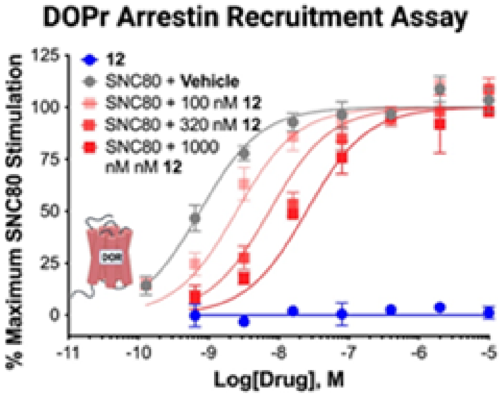

3 Computation

<u>SILCS FragMaps point out key interaction differences between the active and inactive pockets of DOPr :</u> Shown in **Figure S2** are the SILCS FragMaps inside the orthosteric binding site of both the active and inactive forms of the DOPr receptor. The key site in DOPr is adjacent to D128, while apolar site A is formed closer to the key site, and is lined by apolar residues such as L300, I304, Y308 while apolar B is formed mainly by V281, W284 and L300 of TM6, and apolar site C is formed by W274 and H278 (TM6 and TM7) in both the active and inactive pockets of DOPr. As depicted in **Figure S2A**, the apolar sites A and B are connected in the active DOPr pocket forming a long cylindrical apolar region. This is attributed to the cluster of apolar residues including Y129, W274, H278, V281, W284, L300, I304 and Y308 mainly belonging to TM3, TM6 and TM7. Such a cylindrical apolar region is missing from the inactive DOPr receptor pocket (**Figure S2C**), where the residues belonging to TM6 and TM7 are far from each other, also resulting in an extension of the apolar site A (**Figure S2C**). Like the MOPr active pocket, the active DOPr pocket also has an extensive region of acceptor/donor FragMaps that partially overlap with the cylindrical apolar sites A and B. Like the apolar sites in the inactive DOPr pocket, the acceptor/donor site are also located peripherally, towards TM6 and TM7, creating the large acceptor site A. Overall, the apolar and acceptor/donor sites in the active MOPr pocket are more uniformly distributed, with more favorable FragMaps for functional groups such as methanol (MEOO, olive green maps), towards site A, B and

C. The active DOPr pockets have more favorable FragMaps of apolar groups than the active MOPr pockets, while both have similar pattern for acceptor fragments. The inactive DOPr pocket is distinct from the active DOPr pocket as it has a smaller apolar region, due to the diffuse network of apolar residues, opposed to the clustered residues in active DOPr.

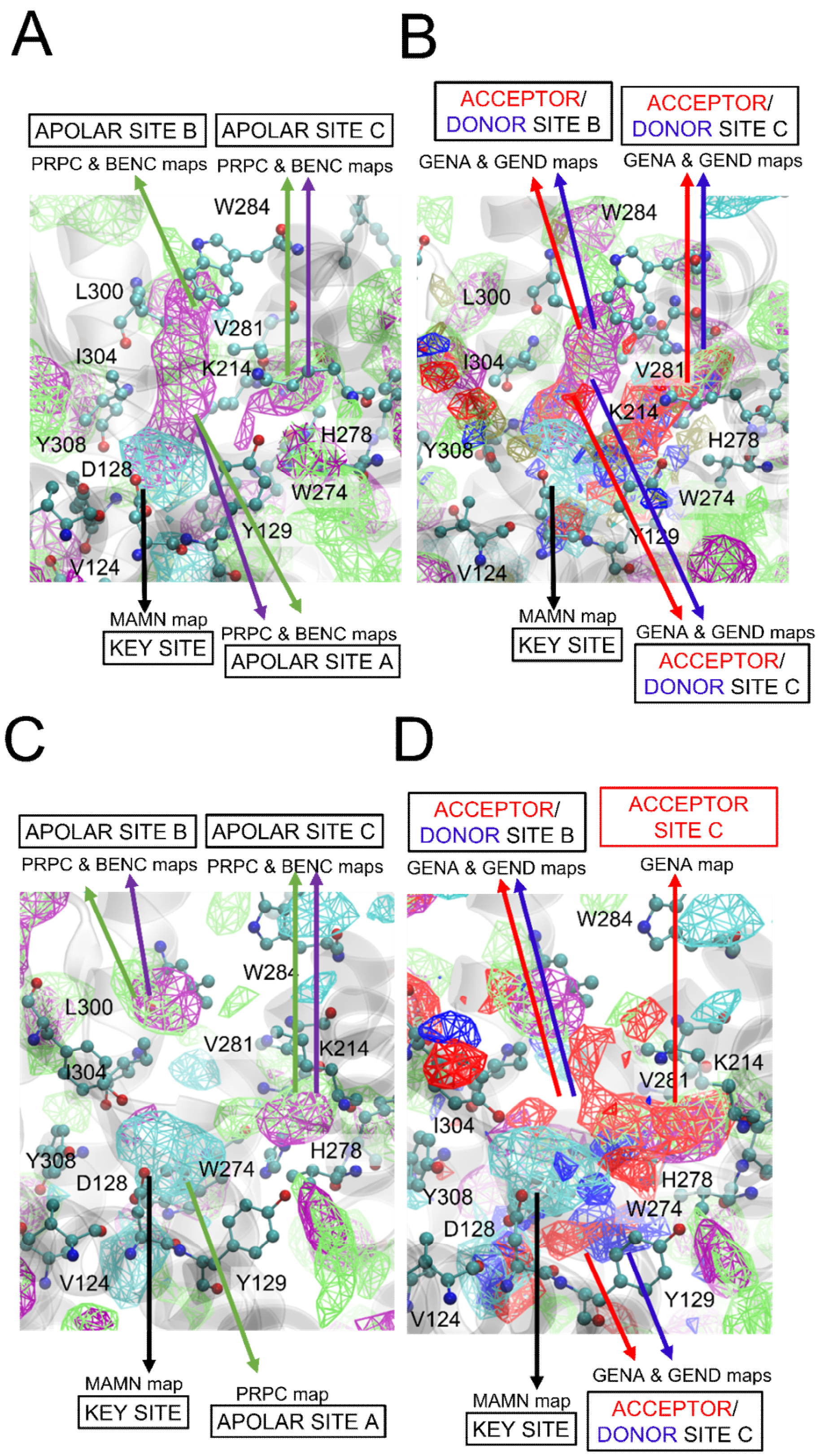

Figure S2: SILCS FragMaps overlaid on the A & B) active and C & D) inactive crystal structures of DOPr. The protein backbone is shown in a transparent gray cartoon with selected sidechains shown in

atom colored CPK format. FragMap Color Code: Benzene (purple), propane (green), methylammonium N positive (cyan), generic hydrogen-bond acceptor (red), generic hydrogen-bond donor (blue), methanol oxygen (olive green), and acetate O negative (orange) at GFE energy contours of -1.2 kcal/mol.

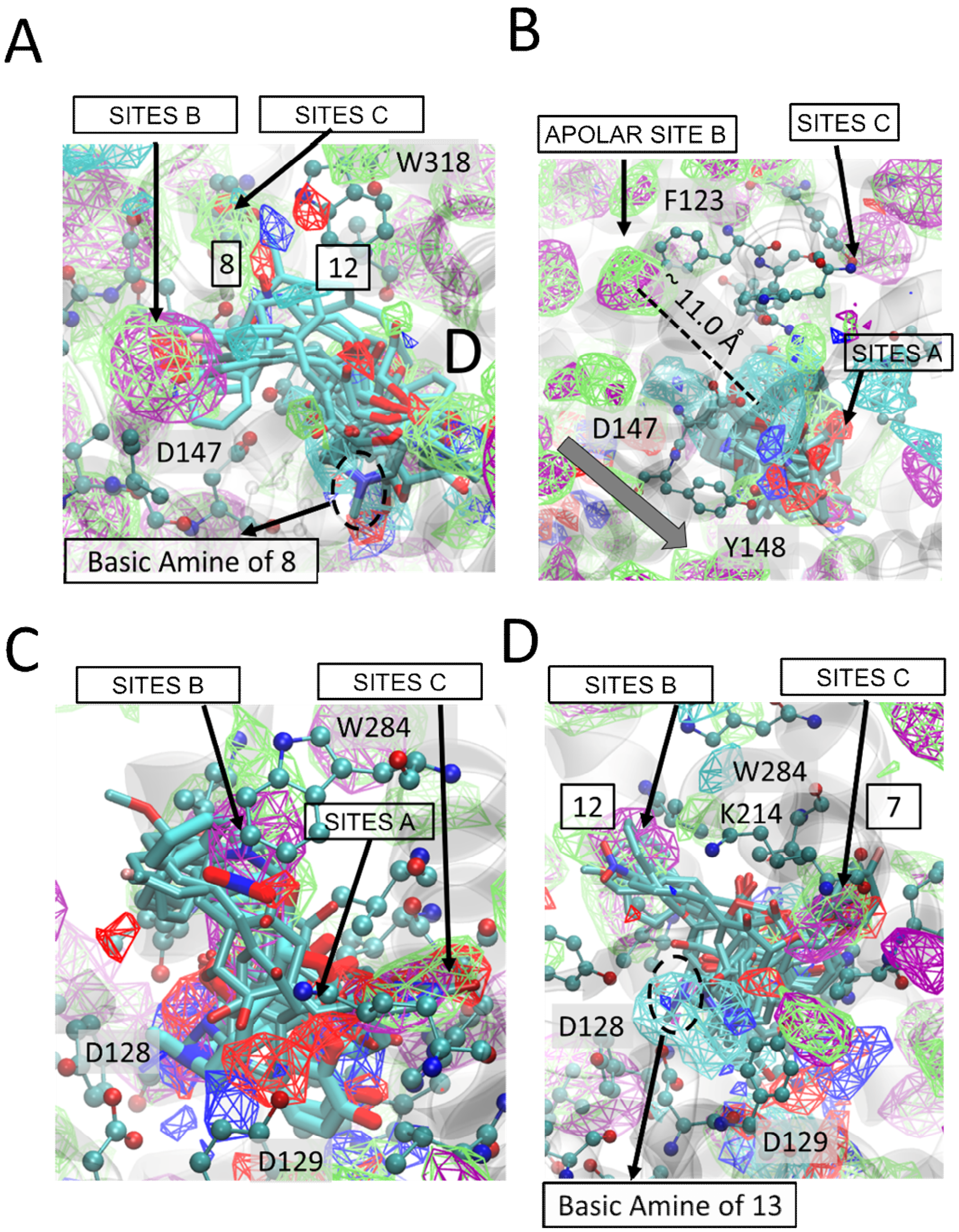

Figure S3. A and **B)** Active and Inactive MOPr; **C** and **D)** Active and Inactive DOPr pockets showing predicted poses of ligands from SILCS-MC docking. Ligands in atom-colored licorice representation and amino acid side chain in CPK representation. FragMap Color Code: Benzene (purple), propane (green), methylammonium N positive (cyan), generic hydrogen-bond acceptor

(red), generic hydrogen-bond donor (blue), methanol oxygen (olive green), and acetate O negative (orange) at GFE energy contours of -1.2 kcal/mol.

As shown in the main text in **Table 3**, the experimental affinities of the top three ligands in the DOPr active form match with the top three SILCS scored compounds. The ranking for in vitro affinity were **12** > **14 ~ 10 ~ 11 > 8**, where the *in silico* SILCS ranking was **14 > 12 > 7** ~ **9 > 8**. While **14** scored the most favorable LGFE score in both DOPr and MOPr, **7** and **9** consistently scored similarly well in both receptors, also assuming near-identical poses. Due to the abundance of the apolar and donor/acceptor maps in the active DOPr, these ligands were approximately -1.0 kcal more favorable than those in the active MOPr. As shown in **Figure S3**, all compounds in the active MOPr and DOPr pockets assumed similar poses, occupying the apolar sites A and B. Exception to this was compound **8** in the active MOPr pocket. As seen in **Figure S3A**, the basic amine of **8** aligns slightly outwards, thus causing its overall misalignment from the apolar region and thus a less favorable LGFE score. **Figure S3B** shows the SILCS-MC poses of ligands in active MOPr receptor, where all ligands, although having their basic amine group close to the key site, are aligned completely out of sites B and C, which led to their less favorable LGFE scores. **Figure S3C and S3D** show all compounds in the active and inactive DOPr pocket, where ligands seated their basic amine group close to the key site, the 4,5-epoxymorphinan core seated in apolar site A, while the head groups seated in apolar pocket B. Despite this, the lack of FragMaps in all the sites of the inactive DOPr, led to the LGFE scores being less favorable by ~2 kcal/mol in inactive DOPr, showing that all test ligands had weaker affinity to inactive DOPr.
